# Role of N-Cadherin *cis* and *trans* Interfaces in the Dynamics of Adherens Junctions in Living Cells

**DOI:** 10.1371/journal.pone.0081517

**Published:** 2013-12-02

**Authors:** Stefanie Bunse, Sakshi Garg, Stephan Junek, Dirk Vogel, Nariman Ansari, Ernst H. K. Stelzer, Erin Schuman

**Affiliations:** 1 Department of Synaptic Plasticity, Max Planck Institute for Brain Research, Frankfurt am Main, Germany; 2 Department of Neural Systems, Max Planck Institute for Brain Research, Frankfurt am Main, Germany; 3 Buchmann Institute for Molecular Life Sciences (BMLS), Goethe Universität, Frankfurt am Main, Frankfurt am Main, Germany; University of Birmingham, United Kingdom

## Abstract

Cadherins, Ca^2+^-dependent adhesion molecules, are crucial for cell-cell junctions and remodeling. Cadherins form inter-junctional lattices by the formation of both *cis* and *trans* dimers. Here, we directly visualize and quantify the spatiotemporal dynamics of wild-type and dimer mutant N-cadherin interactions using time-lapse imaging of junction assembly, disassembly and a FRET reporter to assess Ca^2+^-dependent interactions. A *trans* dimer mutant (W2A) and a *cis* mutant (V81D/V174D) exhibited an increased Ca^2+^-sensitivity for the disassembly of *trans* dimers compared to the WT, while another mutant (R14E) was insensitive to Ca^2+^-chelation. Time-lapse imaging of junction assembly and disassembly, monitored in 2D and 3D (using cellular spheroids), revealed kinetic differences in the different mutants as well as different behaviors in the 2D and 3D environment. Taken together, these data provide new insights into the role that the *cis* and *trans* dimers play in the dynamic interactions of cadherins.

## Introduction

Cell-cell adhesion and signaling is essential for the formation and maintenance of tissues and organs in multicellular organisms. The cadherin superfamily of molecules, characterized by an extracellular domain with multiple, highly conserved repeats, represent critical components of many intercellular junctions. Cadherins are not only important during development, where they play a central role in cell sorting and compartmentalization [1], but they are also ubiquitously expressed throughout the adult human body. Cadherins are usually organized in adherens junctions, points of adhesion between neighboring cells. The so-called classical vertebrate cadherins contain five cadherin repeats that enable a homophilic interaction between molecules emerging from the same membrane (*cis* interaction) as well the opposing membrane (*trans* interaction) [2]. Four Ca^2+^-binding sites for three Ca^2+^-ions each are located between the cadherin repeats. Calcium binding at these sites leads to a rigidification of the extracellular domain that is essential for adhesion. Electron microscopy data on E-cadherin indicate that there is an increase in the rigidification of the rod-like structure as the extracellular Ca^2+^-concentration increases from 0.05 mM to 0.5 mM. At Ca^2+^-concentrations above 1 mM *trans* interactions were observed as well [3]. The basal extracellular Ca^2+^-concentration *in vivo* is 1.2-2 mM [4], which is within the cadherins dimerization range. Cadherins could therefore play an important role as Ca^2+^-sensors, monitoring fluctuations in extracellular Ca^2+^, e.g. at the neuronal synapse, and transferring this information to the inside of the cell via cytoplasmic binding partners. In order to investigate such physiological functions of the cadherins, it is important to understand, in living cells, the *cis* and *trans* binding mechanism of cadherins and its regulation by extracellular Ca^2+^.

**Figure 1:**
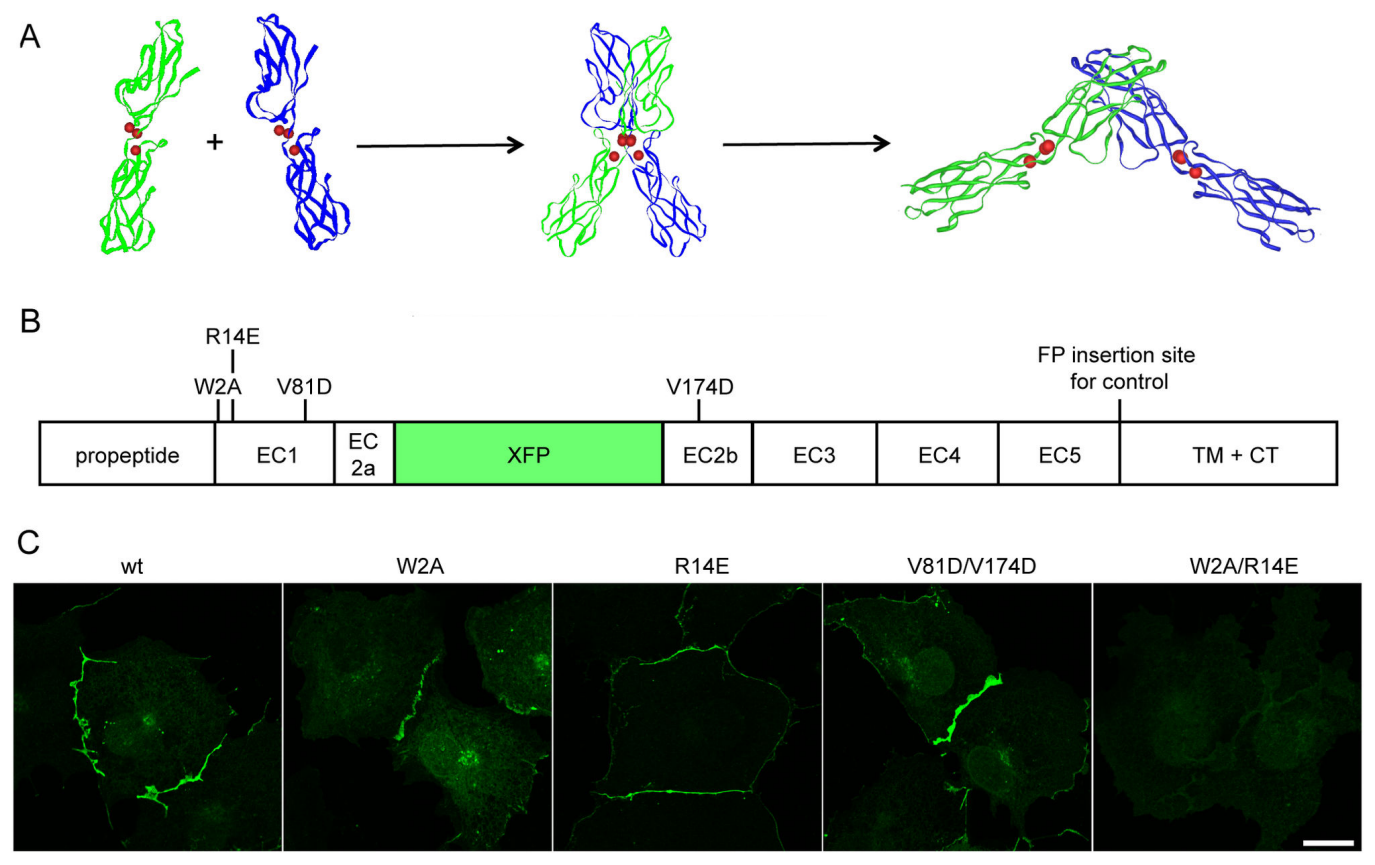
Targeting of *cis* and *trans* binding interface by point mutations. (**A**) Two-step-binding mechanism of cadherin *trans* interaction. Based on crystal structures, a two-step-binding mechanism was proposed for the *trans* interaction of E-cadherin [5]. The structures shown are the two monomers of the EC1-EC2-domain of E-cadherin (PDB 1Q1P) on the left side, the intermediate X-dimer (ECad-W2A, PDB 3LNH) in the middle and the final strand-swapped dimer on the right side (PDB 2QVF). Red spheres indicate the position of Ca^2+^-ions. (**B**) Scheme of fluorescent N-cadherin fusion protein. The insertion of the fluorescent protein (green, XFP; either Venus or Cerulean) into the second cadherin repeat of the extracellular domain of N-cadherin is illustrated. The position of the mutations targeting the *trans* (W2A, R14E) and the *cis* binding interfaces (V81D, V174D) are shown as well as a second insertion site for the fluorescent protein near the EC5 repeat used for control FRET experiments. (**C**) Expression of mutant fluorescent fusion proteins targeting *trans* and *cis* binding interfaces. COS7 cells were transiently transfected with plasmids encoding the N-cadherin-Venus-WT and its binding interface mutants. Besides the double mutant N-cadherin-Venus-W2A-R14E, all fusion proteins were localized at the plasma membrane and formed adhesion junctions. Scale bar 20 µm.

Recently, a mechanism for E-cadherin *trans* binding was proposed based on interfaces identified from the crystal structures of wild-type (WT) and mutant E-cadherin dimers [5]. A so-called “strand-swap” of the tryptophan at position 2 of the mature molecule leads to the insertion of a side chain into a hydrophobic pocket of an opposing cadherin molecule and is essential for *trans* interaction of type-I cadherins (Figure 1 A) [6]. Replacement of this residue by alanine (W2A) prevents the formation of the strand-swapped dimer and leads to the formation of the so-called X-dimer [5] (Figure 1 A - middle image). The X-dimer has been proposed to represent an intermediate step in trans cadherin binding; the lysine at position 14 in E-cadherin is crucial for X-dimer formation. Mutation of lysine 14 to glutamate, however, led to a structure very similar to the WT [5], suggesting that X-dimer formation is not essential for cadherin interactions.

The interface involved in *cis* binding - interaction of the extracellular domains of two molecules on the same cell surface - was recently described based on X-ray crystallographic data. The interface occurs between the base of the EC1 domain of one molecule and a region near the apex of EC2 of a parallel partner [7]. Cadherins expressing a mutation that prevents the *cis* interface can still form the strand-swapped dimer, however, the absence of this interface led to a disruption of the lateral assembly of *trans* dimers into adherens junctions [7]. 

Although the sequence homology between N- and E-cadherin is high, the binding affinities for homodimerization has been shown to be dramatically different - the K_D_ of E-cadherin is many fold higher than N-cadherin K_D_ [8]. Furthermore, the binding specificity between different cadherins is high in favor of homophilic interactions [8]. Thus, understanding the binding mechanism of N-cadherin may differ from what has been shown for the well-studied E-cadherin.

Here we analyze mutants targeting the strand-swapped dimer (W2A), the X-dimer (R14E) and the *cis* interface (V81D/V174D) and find that the described crystal structures for E-cadherin are in part validated for N-cadherin in live cells. We exploited a recently developed Förster resonance energy transfer (FRET)-based platform [9] that monitors *trans* junctional cadherin interactions to study the different binding interfaces for N-cadherin within live cells. The investigation of the Ca^2+^- dependency revealed increased or decreased sensitivities of the mutants compared to the WT. In addition, we used time-lapse imaging of cell lines stably expressing WT or mutant N-cadherin to investigate the role of the molecular interfaces in the assembly and disassembly of cellular junctions. 

Most cell biological studies rely on the use of heterologous cell lines or dissociated cells cultured in a 2D cell culture environment. Many studies [10-12] have shown that cells change their properties with respect to cell morphology, migration, proliferation and adhesion in a 2D environment. *In vivo*, cells connect to each other as well as to the extracellular matrix (ECM), which is rich in proteins like collagen, elastin and laminin that provide biomechanical support, structure and biochemical cues that aid communication between cells [13]. The specific conditions used to maintain cells in 2D cell culture are known to also have a dramatic influence on expression and location of adhesion molecules [14]. Considering these issues, 3D cell culture represents an alternative approach to studying cell biological processes in a more physiological context than maintaining whole organisms [15]. 

Here, we used cellular spheroids [16] to address the relevance of the individual binding interfaces of N-cadherin to cell adhesion in a 3D context. We examined the assembly of cell lines stably overexpressing N-cadherin WT molecules or the mutations W2A, R14E and V81D/V174D into three-dimensional cellular spheroids using light sheet microscopy. Dramatic differences in spheroid assembly were evident in some mutants: while the strand-swap mutant W2A did not form spheroids, the *cis* mutant V81D/V174D formed spheroids only with a considerable delay compared to the WT. Spheroids formed by cells expressing the X-dimer mutant R14E showed an exaggerated necrotic core that was not evident in WT cells.

## Results

We used an N-cadherin-Venus fusion protein [9] to generate N-cadherin mutants corresponding to the binding interface mutants of E-cadherin using site-directed mutagenesis and monitored their localization and dynamics. We created three different mutants that address the significance of the *trans* interaction, the X-dimer formation and the *cis* interaction, respectively. The *trans* interaction was addressed with a strand-swap mutant (mutation that prevents strand-swapping; W2A) [6] and an X-dimer mutant (mutation that prevents formation of the X-dimer; R14E) [5]. We created the X-dimer mutant with the following reasoning: previous studies in E-cadherin had demonstrated that the lysine at position 14 in E-cadherin is critical for the X-dimer interface [5]. As the homology of the ectodomains of E- and N-cadherin is high, we assumed that the positively charged arginine at position 14 in N-cadherin mediates the X-dimer interface. Besides the two single-mutants, a double mutant of W2A and R14E was generated to study the relevance of these mutations for cadherin trans interactions. In order to address the *cis* interface, we generated an N-cadherin double-mutant, changing the valine at positions 81 and 174 to aspartate (V81D/V174D) [7]. The relative positions of the individual mutants are depicted in the scheme shown in Figure 1 B.

We initially transiently expressed either the WT or the mutant N-cadherin-Venus-fusion proteins in the fibroblast cell line COS7 to examine expression levels and the localization of the fusion proteins. The WT fusion protein was localized at the plasma membrane at cell-cell contacts and its expression pattern resembled that reported for native adherens junctions [17,18]. Most of the protein was observed at the plasma membrane and little intracellular fluorescence was evident with the exception of the double mutant W2A/R14E, for which only sparse intracellular signal and no membrane localization was observed. The absence of membrane localization for the double mutant W2A/R14E indicates that at least one of the two interfaces must be present for the formation of adherens junctions (Figure 1 C).

**Figure 2:**
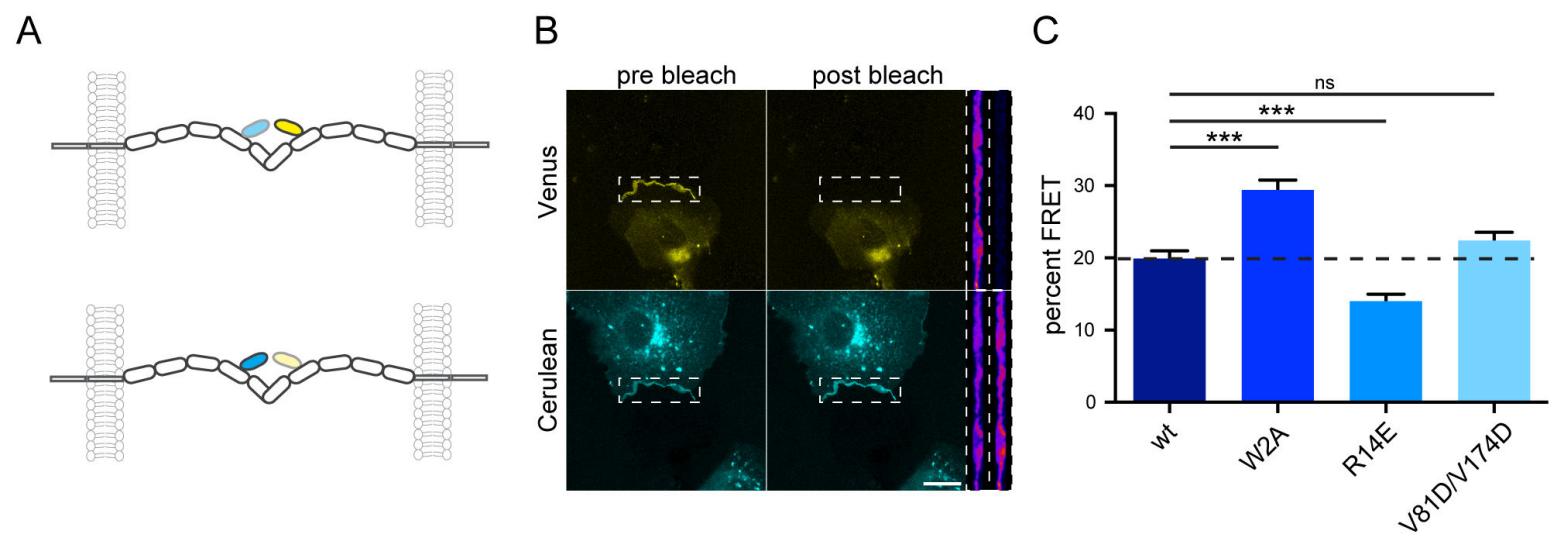
Analysis of the *trans* interaction of N-cadherin in living cells by acceptor bleach FRET experiments. (**A**) Scheme depicting the insertion of Venus (FRET acceptor) and Cerulean (FRET donor) and the interactions of cadherin molecules across a cellular junction. If the two opposing molecules interact with each other in *trans*, the donor and acceptor are within the FRET distance, resulting in an increased acceptor emission and a donor quenching (upper panel). Upon bleaching of the FRET acceptor, the FRET donor is dequenched, leading to an increase in its fluorescence (lower panel). (**B**) Example for an acceptor bleach experiment with COS7-cells expressing either N-cadherin-Venus or –Cerulean. The upper two images show the Venus-channel before and after bleaching of the Venus signal in the junction (boxed region). The Cerulean channel is depicted in the lower two images. Bleaching of the Venus fluorescence leads to a dequenching of the FRET donor Cerulean, which can be observed in the lower two images. An enlargement of the junction (boxed region) is shown next to the images. (**C**) Quantitative comparison of the acceptor bleach experiments for N-cadherin-WT and its mutants. The bars represent the mean ± SEM. The mean of the W2A (n = 32) is significantly increased compared to the WT (n = 35, p < 0.0001, Mann Whitney, ***), while the mean of the mutant R14E was significantly lower (n = 29, p=0.0001, two-tailed unpaired t-test , ***). The *cis* mutant V81D/V174D (n = 33) does not show a significant difference (p=0.1158, two-tailed unpaired t-test). Scale bar = 20 µm.

In order to address the interaction of the above molecules across junctions in living cells, we used a reporter system employing FRET (Figure 2 A), which allows one to monitor cadherin trans interactions with high spatial and temporal resolution [9]. The FRET donor and acceptor comprised N-cadherin molecules in which Cerulean (donor) or Venus (acceptor) was inserted into the second extracellular cadherin repeat (at position 151 of the mature protein). Acceptor bleach FRET experiments were performed to examine the molecular interactions of the WT construct and the different mutants with a high spatial resolution. Two populations of COS7 cells either expressing N-cadherin-Venus or -Cerulean were mixed and plated together at a density appropriate for junction assembly. Bleaching of the FRET acceptor Venus within a junction formed by N-cadherin-Venus and –Cerulean respectively, led to dequenching of the FRET donor Cerulean. The FRET ratio was determined as the ratio (I_postDonor_ – I_preDonor_)/I_postDonor_. In Figure 2 B an example of an N-cadherin-WT acceptor bleach experiment is shown. The mean of the resulting FRET ratio was 19.9 ± 1.1% (mean ± SEM, n = 35) reflecting the *trans* interaction of the N-cadherin-Venus and –Cerulean molecules across the junction (Figure 2 C). The W2A mutant (X-dimer) showed a significant increase (% FRET = 29.4 ± 1.4%, n = 32) in the FRET signal compared to the WT (Figure 2 C; Figure S1). This result confirms our previous data [9] and structural *in vitro* analyses [5] of the W2A mutant showing that the distance of the two EC2 domains is much closer in the W2A mutant than in the WT cadherin. The FRET signal for N-cadherin-R14E (X-dimer mutant) was significantly lower (14.0 ± 0.9%, n = 29) compared to the WT (Figure 2 C; Figure S1). This observation was unanticipated since the crystal structure of E-cadherin-EC1-EC2-K14E, is predicted to be essentially indistinguishable from the E-cadherin-EC1-EC2-WT structure [5]. The FRET efficiency of the *cis* double mutant N-cadherin-V81D/V174D (22.4 ± 1.2%, n = 33) showed no significant difference when compared to the WT indicating that the distance of the EC2 domains of the *trans* dimers did not change due to the *cis* mutation (Figure 2 C; Figure S1). We measured the background signal by conducting an acceptor bleach FRET assay with an N-cadherin clone in which the fluorophore was inserted between the EC5 domain and the transmembrane domain (Figure 1 A). The predicted distance between the fluorophores is ~ 380Å [7], which is well beyond the sensitivity of a FRET experiment. The measured FRET ratio with the EC5-XFP pair was significantly smaller (Figure S1 B, 3.8 ± 2.7%, n = 8) than any of the ratios obtained for the mutant or WT junctions, which indicates that the FRET signal is indeed due to the *trans* interaction of the cadherins.

**Figure 3:**
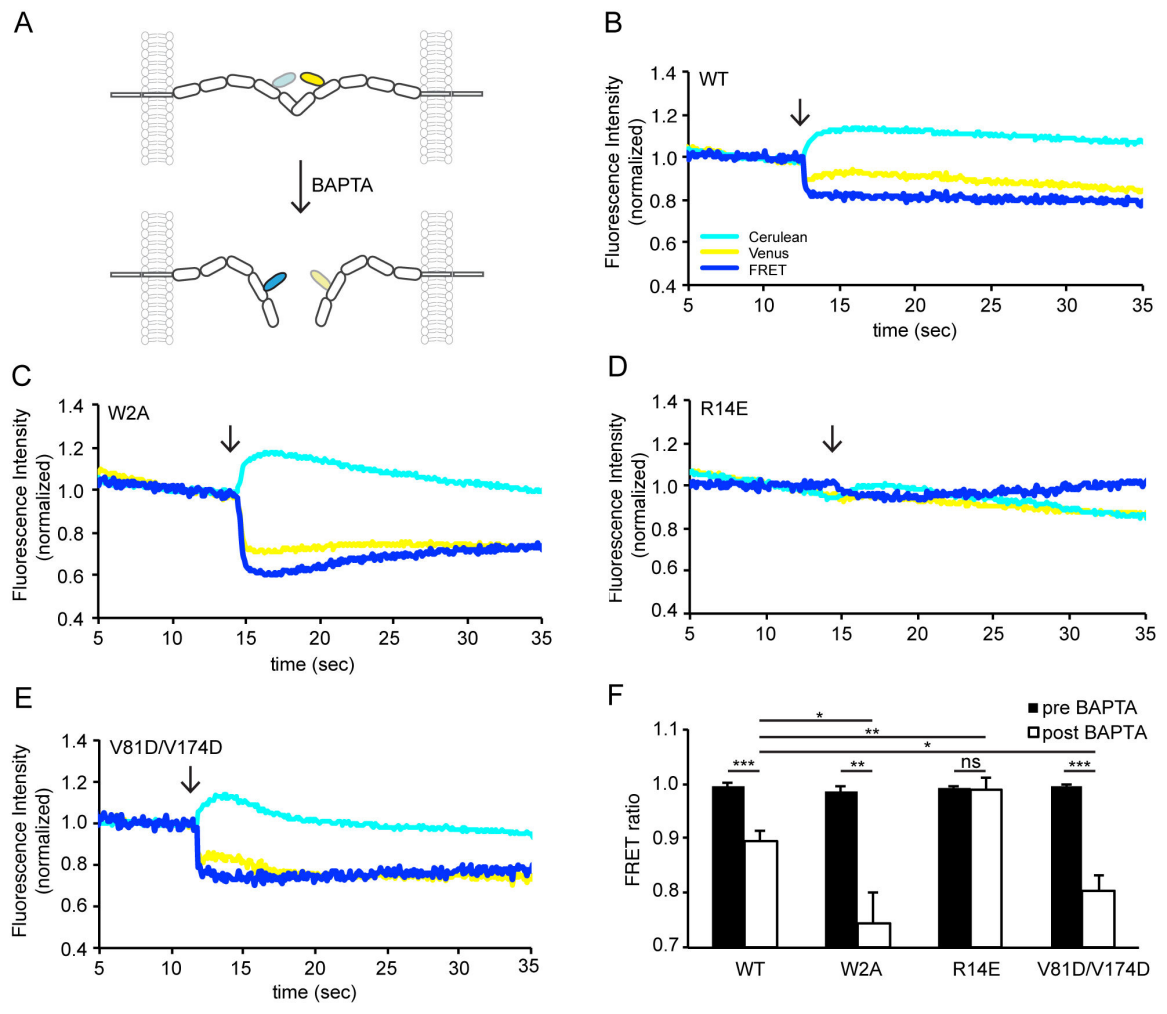
Influence of Ca^2+^-chelation on the individual binding interfaces of N-cadherin. (**A**) Disruption of the *trans* interaction of N-cadherin by BAPTA. Upon disruption of this interaction the fluorophores move further apart, reducing FRET. Examples for ratiometric FRET measurements of the WT (**B**), W2A (**C**), R14E (**D**) and V81D/V174D (**E**) before and after Ca^2+^-chelation are shown. After baseline recording, the Ca^2+^-chelator BAPTA (20 mM final concentration) was added. The fluorescence of the FRET acceptor Venus (yellow) and the donor Cerulean (cyan) as well as the ratio of the two (blue) are shown. (**F**) The quantification of the effect of BAPTA on all mutants is shown. While the mutants W2A and V81D/V174D showed a significantly higher decrease of the FRET signal due to Ca^2+^-chelation than the WT, no Ca^2+^-sensitivity was observed for the X-dimer mutant R14E. This confirms the hypothesis that the X-dimer is a Ca^2+^-dependent intermediate step in the formation of the strand-swapped dimer. n(WT) = 8, n(W2A) = 8, n(R14E) = 9, n(V81D/V174D) = 7 junctions. Statistics were conducted using either paired or unpaired t-test.

The adhesive function of the cadherins is strongly dependent on the extracellular Ca^2+^-concentration [19]. The formation of the intermediate X-dimer interface (represented by the W2A-mutant) has been suggested to be Ca^2+^-dependent [5,20]. Therefore, we tested the Ca^2+^-sensitivity of the individual binding interfaces in living cells by ratiometric FRET measurements before and after the addition of a Ca^2+^-chelator (Figure 3 A). The *trans* interaction was monitored by measuring the ratio of the Venus- to Cerulean-fluorescence over time. Addition of the Ca^2+^-chelator 1,2-bis(o-aminophenoxy)ethane-N,N,N',N'-tetraacetic acid (BAPTA) (final concentration 20 mM) to cells expressing the N-cadherin-WT-junctions led to a rapid and significant decrease in the FRET ratio (Figure 3, B and F, FRET_before_ = 99.6 ± 0.5%, FRET_after_ = 89.6 ±1.9%, mean ± SEM, n = 8) suggesting a partial disassembly of the adherens junction over time [9]. Calcium chelation affected the junctions built by N-cadherin-W2A significantly more than the WT junctions (Figure 3, C and F, FRET_before_ = 98.6 ± 0.8%, FRET_after_ = 74.6 ± 5.4%, n = 8) indicating an increased Ca^2+^-sensitivity of the X-dimer interface compared to the strand-swapped dimer. In contrast, the Ca^2+^-sensitivity was completely abolished in the mutant of the X-dimer, N-cadherin-R14E, which did not exhibit a significant decrease in the FRET ratio after BAPTA addition (Figure 3, D and F, FRET_before_ = 99.2 ± 0.3%, FRET_after_ = 98.8 ± 2.3%, n = 9). Similar to the W2A mutant, the decrease observed for the N-cadherin-V81D/V174D mutant, which abolishes the *cis* interface, (Figure 3, E and F, FRET_before_ = 99.6 ± 0.4%, FRET_after_ = 80.5 ± 2.8%, n = 7) was significantly larger than that observed at WT junctions. This was surprising, as it was not anticipated that the *cis* interface would influence the *trans* interaction. 

**Figure 4:**
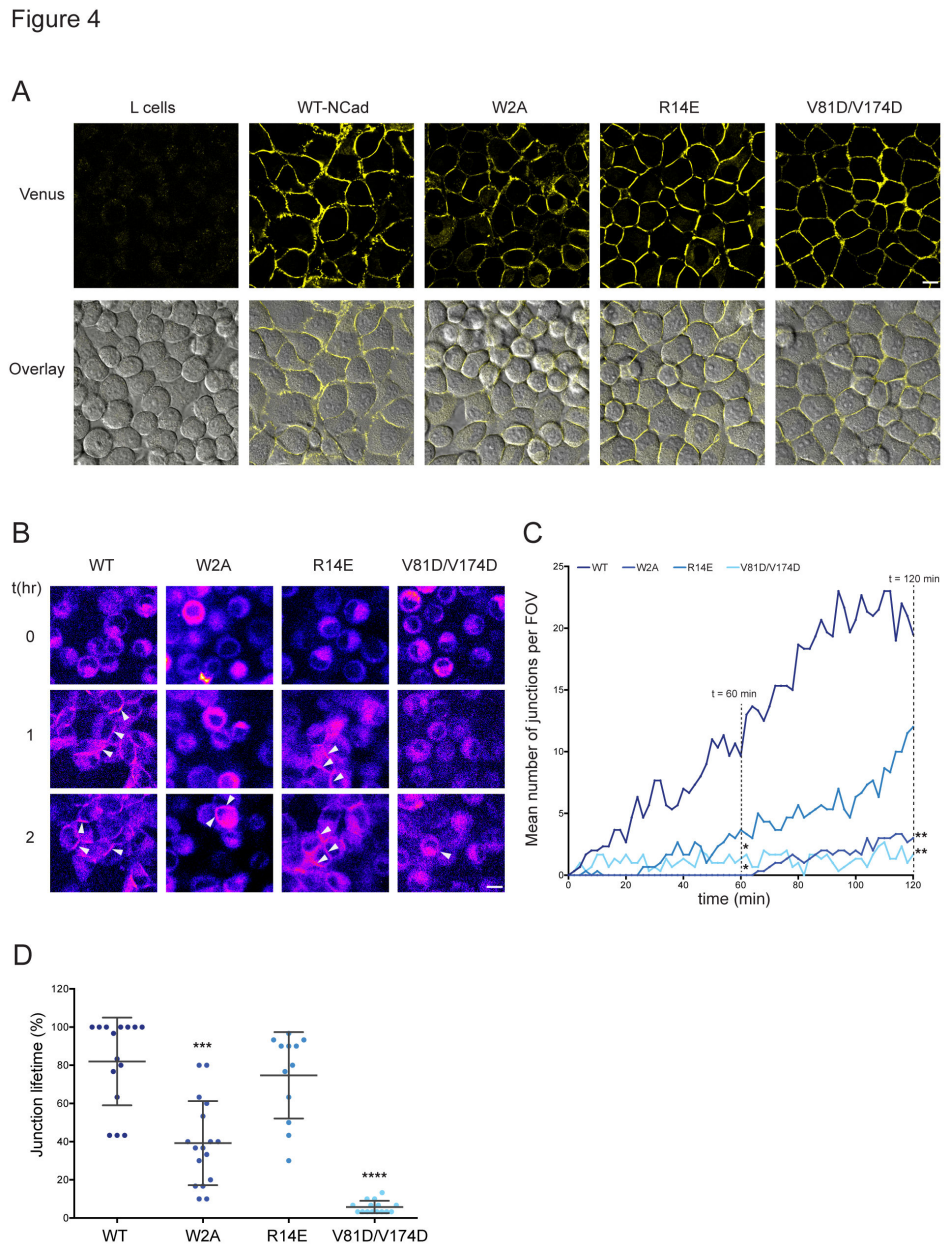
Adherens junction assembly of WT-N-cadherin and its mutants. (**A**) Expression pattern of WT-N-cadherin and its mutants in L cells. Confocal images of control L cells or L cell lines stably expressing the N-cadherin-Venus WT and its mutant forms. Membrane fluorescence can be seen for all mutants demonstrating plasma membrane localization and junction formation for all mutants. Top panel: Venus channel, bottom: Overlay of DIC and Venus channel. (**B**) Example images of junction assembly. Images were taken for all mutants at time 0, 1 and 2 h after imaging commenced. Established junctions can be seen for WT and R14E at 1 h and for all mutants at 2 h (arrowheads). (**C**) Quantification of junction assembly. The number of junctions formed in a field of view (FOV) was counted over time and plotted. The graph shows that the rate of junction formation for WT is fastest followed by R14E. The number of junctions for all mutants was compared to WT at 60 and 120 min. R14E was not significantly different while W2A and V81D/V174D had significantly lower number of junctions at 60 min and 120 min (n = 3 for all mutants, two-tailed unpaired t-test, P < 0.05). (**D**) Quantification of junction lifetime. The junction lifetime for junctions present in a FOV was calculated between 60 and 120 min. On average WT junctions were present for 82% of time, R14E = 75%, W2A and V81D/V174D had significantly lower average junction lifetimes of 39% and 6%, respectively, thus confirming that mutating the *cis* interface leads to highly volatile junctions (Mann-Whitney U test, P < 0.0001). Error bars indicate SD. n(WT) = 15, n(W2A) = 17, n(R14E) = 12, n(V81D/V174D) = 15 junctions. Scale bar (A) and (B) = 10 µm.

We next addressed the role of the individual binding interfaces in the assembly and disassembly of adherens junctions, dynamic processes that occur *in vivo* during the expansion and remodeling of tissue. We used L cell lines stably expressing the N-cadherin-Venus WT and its mutant forms. L cells are mouse fibroblasts, which lack endogenous cadherins [21]. We monitored junction assembly and disassembly processes in live cells using time-lapse imaging (minutes to hours). In the generated stable L cell lines, all mutant N-cadherin proteins were located at the plasma membrane while untransfected L cells (control) did not exhibit any fluorescent signal (Figure 4 A). Long-term live cell imaging was used to monitor the process of junction formation in a humidified, temperature-controlled environment. Cells were dissociated and imaging started 30 min after plating (to allow time for the cells to settle and acclimatize to the incubator conditions). Images were acquired every 2 min for 2 h, using a Spinning Disc microscope, and the presence of junctions was monitored. Example images for the WT and each mutant at multiple time-points are shown in Figure 4 B. No junctions were detected at t = 0 h in any group. The *de novo* assembly of multiple junctions, measured as an increase in membrane fluorescence intensity at the junction between cells, was evident in WT-expressing cells after one hour of imaging. At the same time point (t = 1 h) formation of a few junctions could be observed for the cells expressing N-cadherin-R14E, while no junctions were present for N-cadherin-W2A. The total number of junctions in a field of view (FOV) per frame was counted and plotted over time (Figure 4 C, Movie S1). Mutations within any interface involved in dimer formation (*cis* or *trans*) led to a delay in the rate of junction formation compared to the WT (WT = 0.203 > R14E = 0.095 > W2A = 0.053 > V81D/V174D = 0.006) (Figure S2), suggesting the importance of the different interfaces for the assembly of adherens junctions *in vivo*. The total number of junctions for all the mutants was compared to WT at 60 min (WT = 9.66 ± 2.33, W2A = 0.0 ± 0.0, R14E = 3.66 ± 0.88, V81D/V174D = 1.33 ± 0.66) and at 120 min (WT = 19.50 ± 2.50, W2A = 3.0 ± 0.0, R14E = 12.0 ± 5.0, V81D/V174D = 1.66 ± 0.33). At both time points, the junction formation for W2A and V81D/V174D was significantly reduced relative to WT (P < 0.05). 

To investigate the stability of the junctions formed, the junction lifetime was measured between 60-120 min after junction assembly had commenced (n ≥ 12 junctions for each genotype) (Figure 4 D). The WT junctions were stable and on average present for 82% of the time, while R14E junctions had an average lifetime of 75%. Although the average lifetime of both W2A and V81D/V174D was significantly lower compared to WT, the V81D/V174D-mutation affected junction stability dramatically where the junctions were highly unstable, undergoing constant rearrangement (39% and 6%, respectively, P < 0.0001). 

**Figure 5:**
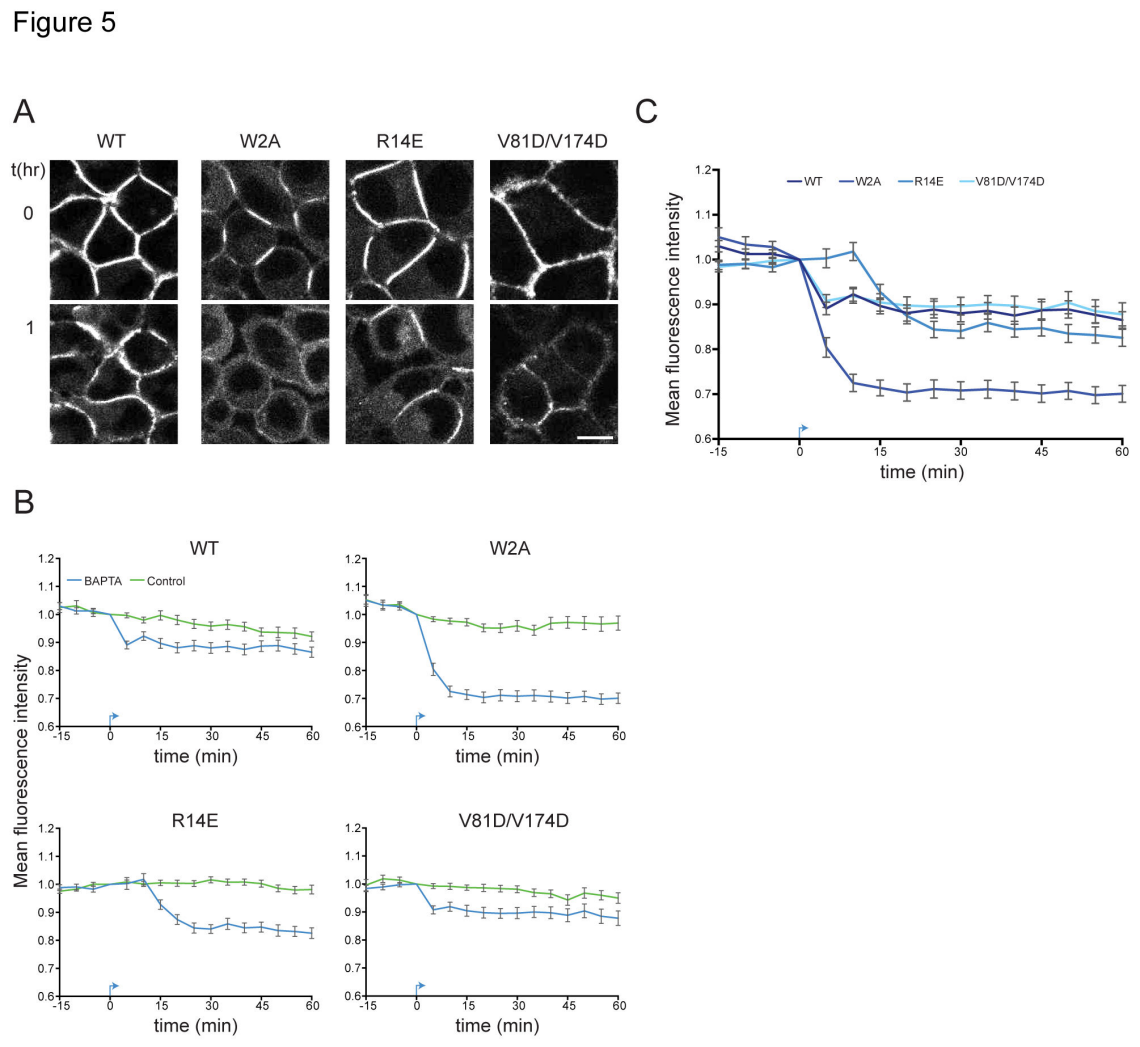
All mutants exhibit sensitivity to prolonged Ca^2+^-chelation. (**A**) Example images for WT and all mutants before and 1 h after BAPTA addition. Scale bar = 10 µm. (**B**) Quantification of junction disassembly. Mean fluorescence intensity of multiple junctions from the different genotypes was measured over time. The drop in mean fluorescence intensity is specific to the addition of BAPTA (blue), indicated by the arrow, as application of a vehicle (green) did not lead to a decrease in junction intensity. (**C**) Comparison of junction disassembly between different mutants and WT. Although the time course and degree of disassembly differed for the various mutants, with W2A junctions displaying the highest degree of disassembly; on a longer timescale, none of them were completely Ca^2+^-insensitive. N-cadherin-R14E, which appeared Ca^2+^-insensitive on a short timescale (Fig. 3 D) also showed a drop in junction intensity 10 min after BAPTA application. Error bars indicate SEM. n(WT) = 84, n(W2A) = 56, n(R14E) = 84, n(V81D/V174D) = 52 junctions.

In order to monitor the disassembly of junctions we examined the effect of Ca^2+^-chelation on adherens junctions formed by the stable L cell lines. Pre-established N-cadherin-Venus junctions were imaged every 5 min using a confocal microscope. After a 15 min baseline recording, 5 mM BAPTA was applied to the cells. Example images for the WT and all mutants before and after BAPTA addition are shown in Figure 5 A. The decrease in mean junctional fluorescence intensity over time was used to quantify the disassembly of junctions (Movie S2). Upon BAPTA addition, an immediate drop in junction intensity was observed for N-cadherin-Venus-WT junctions (𝛕 = 2.63 min) and over a period of 1 h a total 10% loss in junctional intensity was observed. The N-cadherin-Venus–V81D/V174D junctions exhibited a similar decrease in junctional fluorescence intensity following BAPTA addition (𝛕 = 3.54 min) (Figure 5 C & Figure S2). Junctions formed by N-cadherin-Venus-W2A displayed a higher degree of junction disassembly, as indicated by the significantly larger loss in fluorescence. This fits with the FRET experiment data showing that W2A mutant junctions are more sensitive to reductions in extracellular Ca^2+^-concentration than WT junctions (Figure 3 F). Surprisingly, the X-dimer mutant, N-cadherin-Venus-R14E, which appeared Ca^2+^-insensitive on a short timescale in our FRET experiments (Figure 3 D), also showed a drop in junction intensity after 10 min of incubation in BAPTA (𝛕 = 7.28 min). The decrease in junctional intensity for all mutants was specific to BAPTA addition, as application of a vehicle control did not significantly change the junction intensity (Figure 5 B).

**Figure 6:**
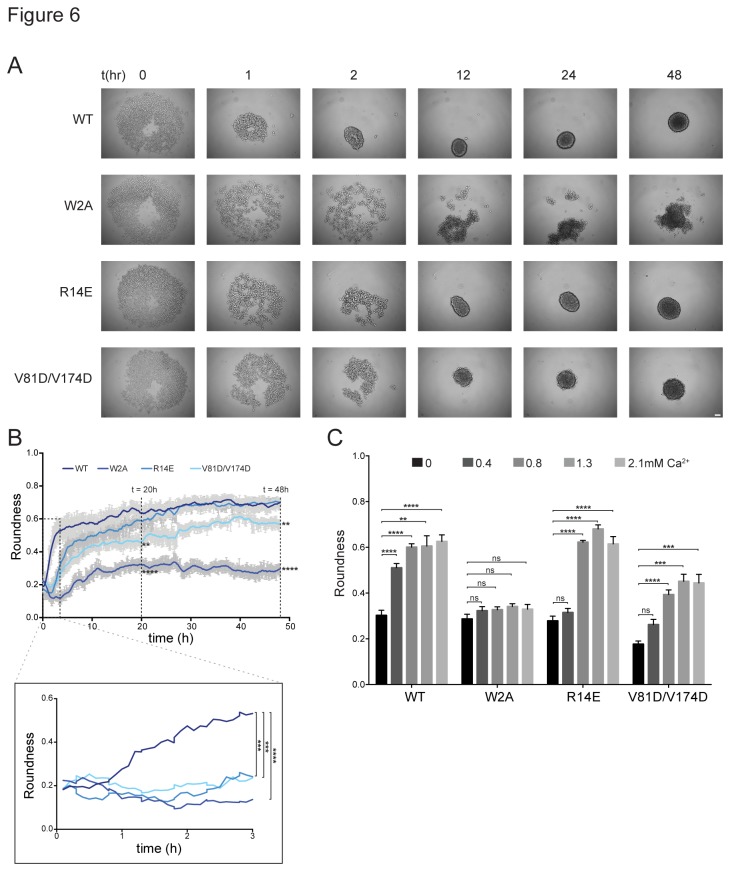
Junction assembly in 3D cell cultures is severely impaired for W2A mutant. (**A**) Example images of spheroid formation at various time points for N-cadherin-WT and its mutants. Scale bar = 50 µm. (**B**) Quantification of spheroid formation. Spheroid roundness was measured over time. At 3 h (inset) the roundness of all mutants was significantly lower than WT. W2A and V81D/V174D had significantly lower roundness even after 20 h and 48 h of spheroid formation (two-tailed unpaired, t-test, P < 0.05). n(WT) = 9, n(W2A) = 9, n(R14E) = 8, n(V81D/V174D) = 8 spheroids. (**C**) Ca^2+^-dependence of spheroid formation. Spheroid formation was monitored in medium with different Ca^2+^-concentrations. The roundness was measured at 20 h after spheroid formation was started. WT-N-cadherin shows a significant increase in roundness with only a slight increase in Ca^2+^-concentration (0.4 mM) and continued to gradually increase. W2A did not show any significant change in roundness regardless of the Ca^2+^-concentration indicating that W2A junctions cannot be rescued with elevated Ca^2+^-concentration. Both R14E and V81D/V174D needed twice the Ca^2+^-concentration (0.8 mM) to show a significant increase in roundness emphasizing that in 3D, loss of either X-dimer or the *cis* interface leads to a decreased Ca^2+^-sensitivity in junction assembly (n for each condition ≥ 4, Mann-Whitney U test, p < 0.05). Error bars indicate SEM.

Next, we investigated cadherin interactions in a tissue-like environment, adopting a 3D cell culture model known as cellular spheroids [22]. Cellular spheroids are formed by the spontaneous aggregation of single cells in to large (100–500 µm) round shaped clusters of cells [16]. Spheroids were formed, using the liquid overlay method [23], with L cells stably transfected with either the WT-N-cadherin protein or one of the mutants (W2A, R14E or V81D/V174D). The formation of spheroids was monitored with long-term live cell imaging, with images captured every 5 min for 48 h (Movie S3). Snapshots of spheroid formation at various time points for the different mutants are shown in Figure 6 A. Formation of spheroids is initiated by cell aggregation, followed by rearrangement and then compaction [24]. To measure the temporal dynamics of spheroid formation, we exploited the simple spherical geometry of the spheroids. The roundness of each spheroid was measured for 48 h and plotted against time (Figure 6 B, n(WT) = 9, n(W2A) = 9, n(R14E) = 8, n(V81D/V174D) = 8). Three time points were chosen to compare the roundness of the genotypes – 3 h, where the WT-N-cadherin spheroid was still in the initial stages of spheroid formation, 20 h, where the WT-N-cadherin spheroid formation had reached a plateau and 48 h, when spheroid formation had stopped. As seen in Figure 6 B, at 3 h, the roundness of all the mutants was significantly lower than the WT-N-cadherin (WT = 0.53 ± 0.06, W2A = 0.13 ± 0.02, R14E = 0.24 ± 0.03, V81D/V174D = 0.23 ± 0.04; P < 0.05), thus indicating that all mutations lead to a delay in spheroid formation. At 20 h and 48 h, R14E was no longer significantly different than the WT-N-cadherin (At 20 h: WT = 0.62 ± 0.02, R14E = 0.61 ± 0.03; At 48 h: WT = 0.68 ± 0.02, R14E = 0.68 ± 0.01; P < 0.05), indicating that although the R14E mutant displayed an initial delay in 3D junction formation, it eventually formed a spheroid similar to WT-N-cadherin. The roundness of W2A and V81D/V174D spheroids was significantly lower than that of WT at 20 h and did not change even after 48 h (At 20 h: W2A = 0.32 ± 0.02, V81D/V174D = 0.44 ± 0.03; At 48 h: W2A = 0.27 ± 0.03; V81D/V174D = 0.57 ± 0.01; P < 0.05). The difference between junction assemblies in 2D versus 3D is quite evident from both W2A and V81D/V174D. In 2D, although delayed, the cells expressing N-cadherin-W2A were able to form a few junctions. In 3D however, the W2A mutant clearly does not form a spheroid. The V81D/V174D junctions were very unstable in 2D and even after 2 h, not many junctions were present. Nevertheless, in 3D, V81D/V174D mutant could form a spheroid, although it was not as round as the WT. The formation of spheroids was further confirmed by the presence or absence of an ECM. Cellular spheroids have been shown to form an ECM consisting of proteins such as fibronectin, laminin and collagen, *in vitro* [25,26]. To determine if an ECM was present in the spheroids formed by the WT-N-cadherin-L cells or one of the mutants (W2A, R14E, V81D/V174D), cryo-sections made through the spheroids were immunostained for laminin. The fluorescence intensity of laminin in the WT, R14E and V81D/V174D spheroids was comparable, whereas in the W2A spheroids the laminin fluorescence intensity was significantly lower, indicating that WT, R14E and V81D/V174D spheroids have an ECM while W2A spheroids do not (Figure S3). 

Given that cadherins are Ca^2+^-sensitive, we monitored spheroid formation in a medium where the extracellular Ca^2+^-concentration was manipulated in a physiological range (0, 0.4, 0.8, 1.3 or 2.1 mM; Figure 6 C). The roundness of the spheroids was measured and compared at 20 h, the time point at which the WT-N-cadherin spheroid reaches a steady state. For WT-N-cadherin, a slight increase in [Ca^2+^]_ext_ from 0 to 0.4 mM was sufficient to significantly increase the roundness of the spheroids (at 0 mM: 0.3 ± 0.02, at 0.4 mM: 0.51 ± 0.02), which gradually increased with increasing [Ca^2+^]_ext_ (at 0.8 mM: 0.6 ± 0.01, at 1.3 mM: 0.61 ± 0.04, at 2.1 mM: 0.63 ± 0.03). For W2A, the roundness of the spheroid did not change regardless of the [Ca^2+^]_ext_, indicating that with long-term imaging in a 3D environment, the W2A junctions cannot be rescued by elevated extracellular Ca^2+^-levels (at 0 mM: 0.29 ± 0.02, at 0.4 mM: 0.32 ± 0.02, at 0.8 mM: 0.33 ± 0.01, at 1.3 mM: 0.34 ± 0.01, at 2.1 mM: 0.33 ± 0.02). For both the R14E and V81D/V174D mutants, an extracellular Ca^2+^-concentration of 0.8 mM (vs. 0.4 mM) was required to see a significant change in spheroid roundness relative to WT-N-cadherin (at 0 mM: R14E = 0.28 ± 0.02, V81D/V174D = 0.18 ± 0.01, at 0.4 mM: R14E = 0.31 ± 0.02, V81D/V174D = 0.26 ± 0.02, at 0.8 mM: R14E = 0.62 ± 0.01, V81D/V174D = 0.04 ± 0.02, at 1.3 mM: R14E = 0.68 ± 0.02, V81D/V174D = 0.45 ± 0.03, at 2.1 mM: R14E = 0.61 ± 0.03, V81D/V174D = 0.44 ± 0.03). This emphasizes that in 3D, loss of either the X-dimer or the *cis* interface leads to a decreased Ca^2+^-sensitivity in junction assembly (n for each condition ≥ 4, P < 0.05). 

**Figure 7:**
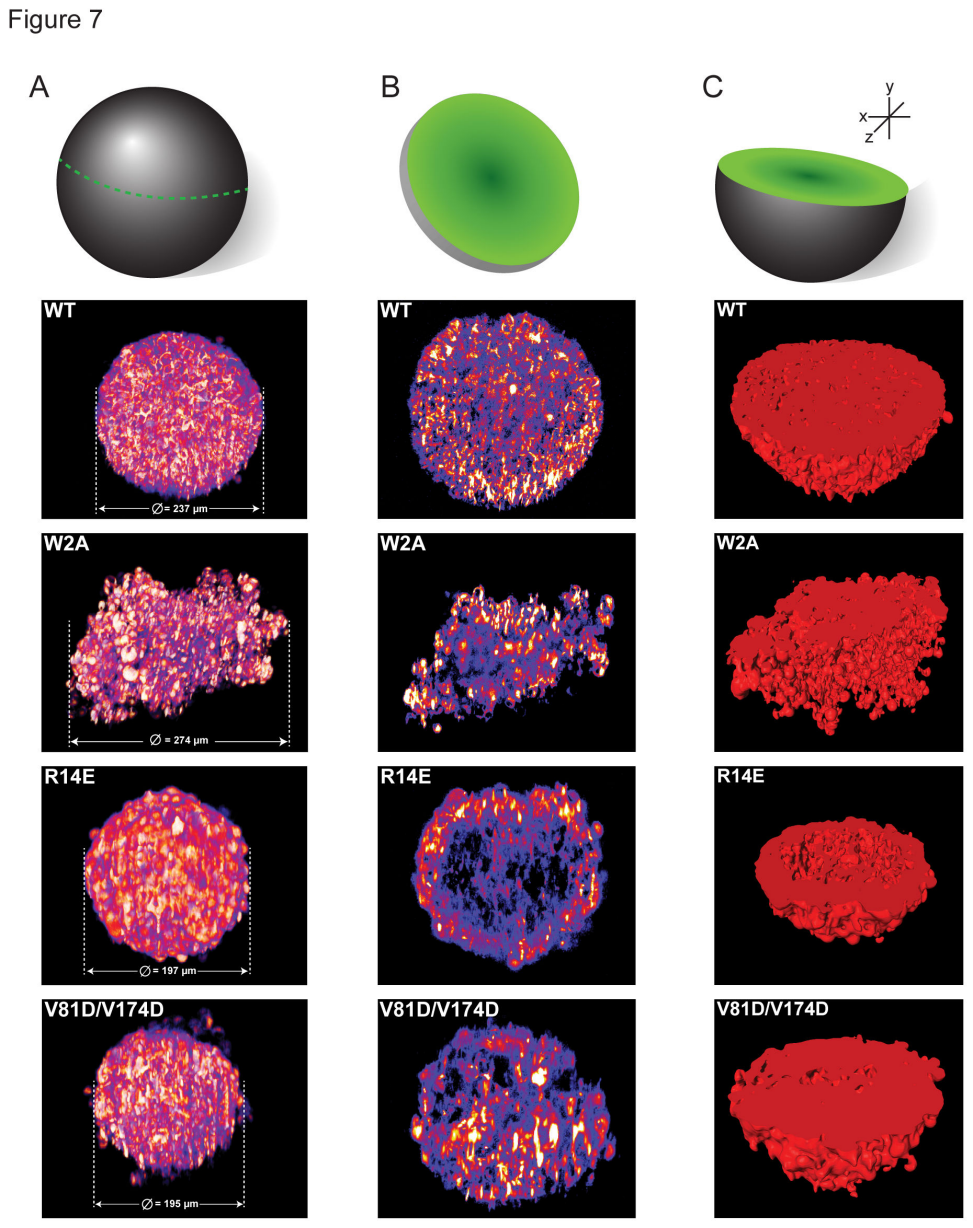
All mutants differ in cellular spheroid organization on global or cellular levels compared to N-cadherin-WT. (**A**) Example images of complete spheroids after fusing the images from 12 different imaging angles. The dramatic difference between WT and W2A is evident, where W2A does not form a cellular spheroid, while WT is an almost circular spheroid. N-cadherin-Venus signal is represented in pseudocolor “Fire” LUT. (**B**) An XY cross-section through the spheroids. A cross-section through the middle of the spheroid highlights the differences in cellular organization between WT, R14E and V81D/V174D. WT had orderly junctions throughout the XY-plane, while R14E was hollow on the inside as seen by the absence of junctions. Although in V81D/V174D junctions were present, they were not organized. (**C**) Surface generation of the spheroids after slicing them into half through XY. If junctions are present, they are represented in red. Compared to WT, the empty space inside R14E is apparent here too. Even though the morphologies of the WT, R14E and V81D/V174D spheroids are quite similar on a global scale, there is a dramatic difference between how the cells are organized within a spheroid highlighting the effect of any mutation on junction assembly in 3D.

Once spheroid formation was completed, we imaged each spheroid with high spatial resolution in order to determine whether there were any morphological differences apparent at the cellular or global levels. As cellular spheroids are thick specimens (hundreds of microns in diameter) and prone to light scattering we used a monolith digital scanned laser light sheet-based fluorescence microscope (mDSLM) [16]. This enabled excellent signal-to-noise ratio, optical sectional ability, large field of view, good spatial resolution, and fast imaging with a very low fluorophore excitation level [27]. Images were obtained by illuminating the sample using a laser light sheet and detecting the fluorescence emission at a 90° angle. The spheroid was rotated at steps of 30° to acquire an overall complete image from all angles. Figure 7 A shows a complete spheroid after fusing the different imaging angles post-hoc (see also Movie S4). The differences in the morphologies of the spheroid between WT-N-cadherin and W2A can be seen, where the WT is an almost circular spheroid while W2A is only a cell aggregate. While R14E and V81D/V174D are almost equal in size globally, inspection of an XY cross-section (Figure 7 B) indicates that R14E is hollow on the inside, lacking junctions, whereas the WT-N-cadherin cross-section has organized junctions throughout the sphere. Although there were junctions present in the V81D/V174D cross-section, they are quite unevenly spaced and not organized. The hollowness of the R14E spheroid was quantified by looking at the relative fluorescence intensity of the junctions in the cryo-sections. Since there were no junctions present in the middle of the spheroid, a significant decrease in the fluorescence intensity was seen for the R14E spheroid in comparison to all other mutants (Figure S3). Figure 7 C is a surface generation of the spheroid after slicing it in half through XY where, if there are any junctions present, they are represented in red. The empty space inside R14E was apparent here too, compared to the WT spheroid. Even though the morphologies of the WT, R14E and V81D/V174D spheroids were quite similar on a global scale, there is a dramatic difference between how the cells are organized within a spheroid: R14E forms a hollow sphere and V81D/V174D forms disorganized junctions.

## Discussion

Three binding interfaces for cadherin-cadherin interactions have been reported thus far: two of them are involved in *trans* interactions, the X-dimer and the strand-swapped dimer, and the third is involved in *cis* interactions. Here, we analysed the functional role of these interfaces in living cells examining their contribution to the Ca^2+^-dependent interactions of cadherins as well as the assembly and disassembly of cell-cell junctions. 

Using point mutations targeting the above interfaces, we demonstrated that fluorescent fusion proteins of N-cadherin WT and its binding mutants R14E and V81D/V174D were able to form adherens junctions. This was demonstrated in two different cell lines, which express (COS7 cells) or lack (L cells) endogenous N-cadherin. This is in contrast to what has been previously shown for the E-cadherin mutant V81D/L175D, which only formed adherens junctions when additional stabilizing point mutations were included and when endogenous cadherins were present as well [7]. Although the sequence homology between the two is high, differences in the molecules N- and E-cadherin, as well as cell type differences could account for the observed differences. Similar to Harrison et al. [7], however, we observed the *cis* junction to be more volatile and transient than the WT junctions. This was also true for the X-dimer W2A, which showed a reduced lifetime of *de novo* assembled junctions. The reported reduced binding affinity of the X-dimer compared to the strand-swapped dimer [5] could be a reason for the higher volatility of this mutant. However, on a long-term scale stable junctions were observed for all mutants except the double mutant W2A/R14E. The double mutant N-cadherin-W2A/R14E did not form adherens junctions suggesting that the mutation R14E, which is similar to K14E in E-cadherin, abolishes the X-dimer interface and that the remaining *cis* interface alone is not sufficient to form adherens junctions [5]. The delay in junction formation observed for all mutations highlights the physiological importance of the various binding interfaces. Interestingly, though the formation was significantly delayed in W2A-mutants, the mutants still eventually formed junctions indicating that the X-dimer interface is sufficient for junction assembly. 

We used a FRET-reporter system to analyze the mutated binding interfaces that were previously identified by crystallography [5,7]. As before [9], we observed an increased FRET efficiency for the W2A junctions compared to the WT junctions, consistent with the X-dimer structure obtained for E-cadherin by crystallography [5] showing that the EC2 repeats are in closer proximity in the W2A mutant when compared to the WT molecule. However, the structure of the EC1-EC2 domains of the X-dimer interface mutation in E-cadherin-K14E was reported to be nearly identical to the corresponding WT structure [5]. The significantly decreased FRET ratio for the N-cadherin-R14E suggests the EC2 repeats are further apart in the R14E mutant than in the WT and thus the overall molecule is presumably less curved than observed in the crystal structure of E-cadherin-K14E. Since no crystal structure for N-cadherin-R14E has been published so far, it could be that the elimination of the X-dimer interface has more severe effects in N-cadherin than in E-cadherin. Alternatively, the structure of N-cadherin in living cells may deviate from that observed in crystal structures. 

The Ca^2+^-dependence of the different binding interfaces was addressed by ratiometric FRET measurements, in which the FRET efficiency was measured over time and the response to Ca^2+^-chelation was analyzed. The W2A (X-dimer) and the V81D/V174D mutants (*cis* interface) showed a stronger sensitivity in the FRET ratio compared to the WT, while the X-dimer mutant R14E was insensitive to Ca^2+^-chelation in these short-term experiments. This implies that the X-dimer interface is the only Ca^2+^-sensitive interface: the FRET efficiencies of the W2A mutant, which possesses both the X-dimer and the *cis* interface, and the V81D/V174D mutant, which still possesses the X-dimer and the strand-swap interface, both exhibit similar decreases, while loss of the X-dimer interface (R14E) leads to Ca^2+^-insensitivity. The decrease in the FRET efficiency of the WT junctions was significantly smaller than that observed for W2A and V81D/V174D, which may indicate the presence of two populations of cadherin trans dimers, one having already performed the strand-swap and therefore insensitive to Ca^2+^-chelation, and a second one present in the X-dimer state, and therefore sensitive to Ca^2+^-chelation. Due to the rapid basal turnover of N-cadherin [28], it is likely that at a given time point a substantial fraction of cadherin molecules are newly inserted into the membrane. The X-dimer has been proposed to be a fast, intermediate step in the formation of the strand-swapping process, while the final strand-swapping step is thought to be slower. Therefore, we propose that a “non-negligible” fraction of cadherins present at junctions can be found in the X-dimer configuration. 

The role of the X-dimer in establishing *trans* interaction has been established [5], however, our assembly assay confirms that the X-dimer interface is not absolutely essential for adherens junction assembly. We observed that *de novo* junction assembly was possible for the X-dimer mutant R14E, even though it was delayed compared to WT. This indicates the presence of an additional junction assembly pathway that may bypass the X-dimer leading to the strand-swapped dimer. 

Since previous work has shown that the expression of adhesion molecules differs in cells grown in 2D versus 3D, we further investigated junction assembly with WT-N-cadherin and its mutants in 3D using cellular spheroids [16]. Two previous reports suggested that X-dimer mutant (R14E) cannot form cell aggregates in aggregation assays [5,29]. In our 3D spheroid experiments we observed that the R14E cells formed spheroids, evident 20 h after plating, though spheroid formation was delayed relative to WT. This is in contrast to the 2D aggregation assay studies reported thus far in which the R14E mutant does not aggregate even after 24 h [5,29], highlighting the difference between 2D versus 3D approaches. Although spheroids formed by the X-dimer mutant R14E reached the same roundness as the WT, light sheet microscopy revealed dramatic differences in the morphology of the R14E spheroids. While the WT spheroids exhibited regularly arranged junctions throughout the spheroid, the R14E spheroids were lacking N-cadherin junctions within the core of the spheroid. This kind of spheroid is reminiscent of spheroids formed by endothelial cells or epithelial cell lines like MDCK and mammary epithelial cells that form a spheroid with a hollow center as a result of apoptosis/necrosis of central polarized cells [30,31]. It is tempting to speculate that perhaps the R14E mutation leads to polarization of the L cells via the ß-catenin signalling pathway and hence forms a spheroid with a lumen [16,24]. 

In addition to the R14E mutation, the mutations of the *cis* interface, V81D/V174D, and of the strand-swapped dimer, W2A, had a strong impact on the morphology of the spheroid, which could not have been elucidated with a regular two-dimensional culture system. The ablation of the strand-swapped dimer (W2A mutant) completely disabled spheroid formation as shown by the roundness analysis and the decrease of laminin staining, an indicator for the absence of an ECM. This is in line with previous aggregation assays, showing that the “adhesiveness” is mediated by the strand-swapping of the tryptophan residue [5,9]. However, it also demonstrates the importance of three-dimensional cell culture, as this severe phenotype could not have been anticipated by the morphology and behaviour of the adherens junctions in a two-dimensional context. In contrast to the W2A mutant, the V81D/V174D did form spheroids, as shown by the extracellular laminin, but it did not reach the same roundness as the spheroids formed by the WT. Furthermore, the junctions inside the spheroid appeared less ordered compared to the WT. The high-volatility observed during the 2D junction assembly experiments with V81D/V174D might explain why the morphology was altered and the spheroid formation delayed.

Our results shed new light on the role the of the various N-cadherin binding interfaces in cell-cell junctions assembled in two- and three-dimensional contexts in living cells. In addition, we elucidate the interaction of these interfaces with the dynamics of [Ca^2+^]_ext_. The role of extracellular Ca^2+^-signaling has been shadowed by many years of extensive work done on understanding the signaling by intracellular Ca^2+^. There is, however, accumulating evidence that extracellular Ca^2+^-concentration is dynamic and fluctuations have been shown to occur in cardiac muscle [32], islets of Langerhans [33] and neuronal synapses [34]. Due to the localization of N-cadherin at neuronal synapses and other cell-cell junctions and its ability to rapidly react to changes in extracellular Ca^2+^-concentration, it is of particular interest as a sensor of physiological fluctuations in extracellular Ca^2+^-concentrations. 

Mutations or altered expression levels of cadherins are associated with several cancers [35-37]. For example, loss of E-cadherin results in diffuse tumors invading surrounding tissues as single cells [38] and an increase in N-cadherin expression promotes tumor cell survival, migration and invasion [39]. Given the role of N-cadherin in synaptic development and neural circuit formation [40], it is not surprising that alterations in cadherin function have been implicated in a variety of neurodevelopmental and neuropsychiatric diseases [41,42]. Indeed, with the increased availability of data from genome-wide association studies cadherin family members have been associated with a large number of disorders including epilepsy, autism, bipolar disorder and schizophrenia (Redies et al., 2012). Given the large extracellular portion of N-cadherin it is reasonable to speculate that a substantial fraction of the above dysfunction arises from perturbation of the cadherin interfaces, leading to alterations in cadherin organization and adhesion. 

## Materials and Methods

### FRET assay

#### Acceptor bleach FRET

12bit-images with a size of 512 x 512 pixels (106.07x106.07 µm) were acquired with a confocal laser scanning microscope LSM780 (Carl Zeiss, Inc.) using the spectral imaging “lambda mode”. A Plan-Apochromat 40x/1.4 oil objective (Carl Zeiss, Inc.) and the 458nm line of an argon laser were used to obtain an optimal excitation of both fluorophores. Spectral detection occurred from 473nm to 569nm with a bandwidth of 8.7nm. Individual spectra were obtained from adherens junctions of cells expressing the same N-cadherin fluorescent fusion protein. Spectra were saved to the databank and used to unmix images taken from cells that express the two different fluorescent fusion proteins using the “linear unmixing”-function of the Zeiss software ZEN. After the acquisition of a cell pair image expressing different fluorescent fusion proteins the region of the adherens junction was marked by a rectangular region of interest (ROI) and the Venus signal within this ROI was bleached with high laser power (20 iterations, 100% laser power, 514nm). This bleaching was followed by the acquisition of a second image and the subsequent unmixing of both images. 

#### Ratiometric FRET

Kinetic measurements and the Ca^2+^-dependence of the WT and the mutant junctions were addressed by ratiometric FRET measurements. Time-lapse images of a ROI containing the adhesion junction formed by one N-cadherin-Venus- and one N-cadherin-Cerulean-expressing cell were obtained in the lambda mode (same settings as for the acceptor bleach experiments, see above) with a time interval of 100 ms for 35 sec. The Ca^2+^-chelator BAPTA was added to the dish up to a final concentration of 20 mM after a baseline recording (10-15 sec). Images were unmixed using spectra acquired from single-colored junctions. 

### Live cell imaging

#### Junction assembly

N-cadherin-L cells (wt: [9], for generation of mutant lines see supporting information) were split prior to imaging, counted and 3 x 10^6^ cells, resuspended in Hibernate A low fluorescence buffer (BrainBits, LLC), were added to a 35 mm MatTek dish (MatTek, Corp.). The dish was immediately placed in the incubator that was set to 37°C and 5% CO_2_ on the spinning disc microscope BD Pathway 855 (BD Biosciences) and allowed to acclimatize for 30 min. A z-stack (16-bit 672 x 512) of 6 images was captured every 2 min for 2 hours using 40x 0.90 N.A. air Apochromat objective lens (Olympus), 514 nm excitation and an Orca ER-1394 (Hamamatsu, Corp.). 

#### Junction disassembly

N-cadherin-L cells were plated in 35mm MatTek dishes (MatTek, Corp.) so that all mutants had similar cell densities on the day of imaging. Cells were allowed to form junctions over a period of 24 - 48 hours. Prior to imaging, cell nuclei were stained with Hoechst 33342 (Sigma Aldrich) for a minute. Cells were imaged in Hibernate A low fluorescence buffer (BrainBits, LLC). The cells were allowed to acclimatize inside a custom-built chamber heated to 30°C mounted on a LSM 780 (Carl Zeiss, Inc.) microscope for 30 min before imaging. A 40x 1.4 N.A. oil immersion plan-Apochromat objective lens (Carl Zeiss, Inc.) was used to acquire a z-stack (16-bit 1024 x 1024) consisting of 6 z-planes every 5 min using 514nm and 405nm argon ion laser excitation. After 15 min of baseline imaging, 5 mM BAPTA (Alfa Aesar) was pipetted into the dish and imaging was continued for 1-hour post treatment. A final concentration of 5 mM BAPTA was chosen for time lapse imaging because on a longer time scale 20 mM BAPTA led to cells detaching from the dish. 

### 3D cell culture and imaging

L cells were split and then counted. 2000 cells were added to a well in a coated HydroCell 96-U well dish (Nunc) (to avoid cells sticking to the bottom of the dish). Normal growth DMEM medium (supplemented with 10% FCS, 1% sodium pyruvate and 600 µg/ml G418) was used for spheroid formation. For the Ca^2+^-assay, Ca^2+^-free DMEM (including all the other supplements) was used in order to remove extracellular Ca^2+^ and adjust the Ca^2+^-concentration to 0, 0.4, 0.8, 1.3 or 2.1 mM using CaCl_2_. At 0 mM Ca^2+^, Ca^2+^-free serum (Labtech, Ltd.) was used to avoid effects that background Ca^2+^ may have on spheroid formation. The dish was centrifuged for 2 min at 300 g to bring all the cells to the center of the well and was placed in the Cell Observer (Carl Zeiss, Inc.) with the incubator set to 37°C and 5% CO_2_. After determining the position of each well, image acquisition commenced. Images were captured every 5 min for 48 hours using a 5x 0.16 N.A. plan-Apochromat objective lens (Carl Zeiss, Inc.), transmitted light and an AxioCam MRm (Carl Zeiss, Inc.). The position of the well was updated periodically if the spheroids moved out of the field of view. 

### Light sheet-based fluorescence microscopy

48 h post spheroid formation, the spheroids were fixed in freshly prepared 4% PFA in PBS at 4°C for 30 min. The cell nuclei were stained with Hoechst 33342 for 2 h at room temperature. The spheroids were then embedded in 1% low-melting agarose and drawn into a 20 µl Brand intra MARK micropipette according to the method described in Pampaloni et. al [15]. The micropipettes were placed in a suitable sample holder and mounted vertically into the LSFM in a designated perfusion chamber filled with 1x PBS (Gibco). Prior to imaging, the spheroid surrounded by agarose was extruded from the micropipette. The samples were illuminated by a 2.5x 0.06 N.A. Epiplan-Neofluar air objective lens (Carl Zeiss, Inc.) and emitted light was detected by a 10x 0.3 N.A. water immersion N-Achroplan objective lens (Carl Zeiss, Inc.) using the Andor Clara camera (Andor Technology PLC). The sample was excited using a 405 nm and a 488 nm diode laser line (Omnicron GmbH) for Hoechst and Venus, respectively. Three-dimensional recordings were generated by moving the sample along the z-axis – towards the detection lens [16]. In order to capture a complete representation of the spheroid, images were acquired from twelve different angles by rotating the sample along the y-axis, with consecutive angles 30° apart (Figure S4 D). 

## Supporting Information

Figure S1 **Examples of acceptor bleach experiments for mutants and analysis of control acceptor bleach experiments.** (**A**) Examples for acceptor bleach experiments with COS7-cells expressing either N-cadherin-FP-W2A (strand-swap mutant), -R14E (X-dimer mutant) or -V81D/V174D (*cis* mutant). The upper two images show the Venus-channel before and after the bleaching of the Venus signal in the junction (boxed region). The Cerulean channel is shown in the lower two images. Bleaching of the Venus fluorescence leads to a dequenching of the FRET donor Cerulean, which can be observed in the lower two images. An enlargement of the junction (boxed region) is shown next to the images. Scale bars = 20 µm. (**B**) Quantitative comparison of the acceptor bleach experiments for N-cadherin-WT with the insertion of the fluorescent protein within EC2 (WT) or close to EC5 (EC5). The bars represent the mean ± SEM. The mean of the WT (n = 8) is significantly increased compared to EC5 (n = 8, p = 0.0003, unpaired t-test, *** ).
(TIF)

Figure S2 **Curve fitting for junction assembly and disassembly.** (**A**) A linear function following a constant baseline was fit to the junction assembly data and the slope was used to determine the rate of junction assembly. WT has the fastest rate of junction formation, followed by R14E, W2A and V81D/V174D. (**B**) An exponential fit for temporal dynamics of disassembly was performed from the time point of BAPTA addition. The fit for R14E was started 10 min after BAPTA addition, the time point at which the decay started.
(TIF)

Figure S3 **Curve fitting for spheroid formation and analysis of cryo-sections.** (**A**) A sum of two exponentials – fast and slow – following a constant baseline were fit to the roundness data up to 20 h. The tau is displayed for the fast exponential. WT has the fastest rate of spheroid formation, followed by R14E, V81D/V174D and W2A. (**B**) 10 µm cryo-sections of the spheroids were imaged with 20x 0.8 N.A. Plan-Apochromat objective lens. Using a 99-pixel wide line, each section was straightened along 3 axes, distance binned, and relative fluorescence intensity profiles were plotted. Comparing the intensity profiles between bins 30-50 for all the mutants, only R14E was significantly different compared to WT. (Kruskall-Wallis ANOVA with Dunn’s multiple comparison test, P < 0.05). Error bars indicate SEM. n(WT) = 18, n(W2A) = 20, n(R14E) = 25, n(V81D/V174D) = 18 straightened sections. (**C**) The cryo-sections were immuno-labeled using an anti-laminin antibody. Using automated detection of laminin on the periphery of the sections, the mean fluorescence intensity of laminin staining was measured. The mean fluorescence intensity of W2A was significantly lower than WT (Mann-Whitney U test, P < 0.0001). Error bars indicate SEM. n(WT) = 7, n(W2A) = 5, n(R14E) = 6, n(V81D/V174D) = 7 spheroids. Scale bars (B) and (C) = 50 µm.
(TIF)

Figure S4 **Analysis pipeline and 3D imaging illustration.** (**A**) Identification of junction location across time series. (**A1**) Maximum intensity projection of two central images of z-stack (red: Venus, green: Hoechst) at time point -15 min. (**A2**) Threshold mask of Venus channel. (**A3**) Cell centers (yellow dots) calculated based on a watershed segmentation (blue lines). The watershed was calculated in 3D (x,y,t), so that the ‘identity’ of the cells was preserved across the whole time series. (**A4**) Two-closest neighbors’ segmentation: Each color patch represents the pixels that share the same pair of cells as their closest neighbors (based on the cells’ centers shown in A3). (**A5**) Masking segmentation based on distance from the closest cell center. (**A6**) Combination of the threshold mask (A2) with the two-closest neighbor segmentation (A5). Since cell identity is preserved across frames, the junction identity is too, making it possible to track junctions even when they are discontinuous in space and/or time. (**B**) Examples of segmentation across time. The top row (B1-B3) shows the fluorescence images (similar to A1) at time points 0, 15 and 30 min. The bottom row (B4-B6) shows the corresponding segmentation (similar to A6). Note that junctions preserve identity (color) across frames. Segmentations that do not represent individual (e.g. star in B5) or complete (e.g. arrow heads in B4) junctions were excluded from further analysis. (**C**) Identification of spheroid formation over time. Raw data shows the image as it was acquired. An Ilastik classifier was used to define two classes – spheroid (green) and background (red). The pixel segmentation based on the classifier followed by object detection based on the size of spheroid-classified pixels. (**D**) Imaging the spheroids with mDSLM. The spheroid (yellow) was illuminated (blue arrow) from the side and the detection was done at a 90° angle (green arrow). The spheroid was rotated 12 times in steps of 30° as illustrated.
(TIF)

Methods S1 **Supporting materials and methods.**
(DOC)

Movie S1 **Junction assembly of N-cadherin-Venus-WT L cells and its mutants W2A, R14E and V81D/V174D.** L cells stably expressing N-cadherin-Venus WT and its mutants were used to monitor junction assembly. Cells were imaged every 2 min for 2 hours using a BD Pathway 855 spinning disc microscope (BD Biosciences).
(MOV)

Movie S2 **Junction disassembly of N-cadherin-Venus-WT L cells and its mutants W2A, R14E and V81D/V174D.** Cells with pre-established junctions were used to monitor junction disassembly following addition of 5mM BAPTA. Baseline images were captured every 5 min for 15 min, followed by drug addition and then imaging was continued for 1 hour. Images were acquired using LSM 780 microscope with an incubator at 30°C (Carl Zeiss, Inc.).
(MOV)

Movie S3 **Spheroid formation with N-cadherin-Venus-WT L cells and its mutants W2A, R14E and V81D/V174D.** The cells were added to a 96-U well dish and spheroid formation was monitored using the Cell Observer (Carl Zeiss, Inc.) by acquiring images in transmitted light every 5 min for 48 hours.
(MOV)

Movie S4 **3D rendering of spheroids imaged using a light-sheet microscope.** The N-cadherin-Venus-WT and the mutant spheroids were imaged using the mDSLM from 12 different angles, which were combined post-hoc in Amira (FEI Visualization Sciences Group). A volume rendering was used for visualization of the 3D spheroid.
(MOV)
