# Supplementary figures and images for: Role of N-Cadherin cis and trans Interfaces in the Dynamics of Adherens Junctions in Living Cells
Source: PLoS One. 2013 Dec 2;8(12):e81517. doi: 10.1371/journal.pone.0081517 (PMC3847041; doi:10.1371/journal.pone.0081517)

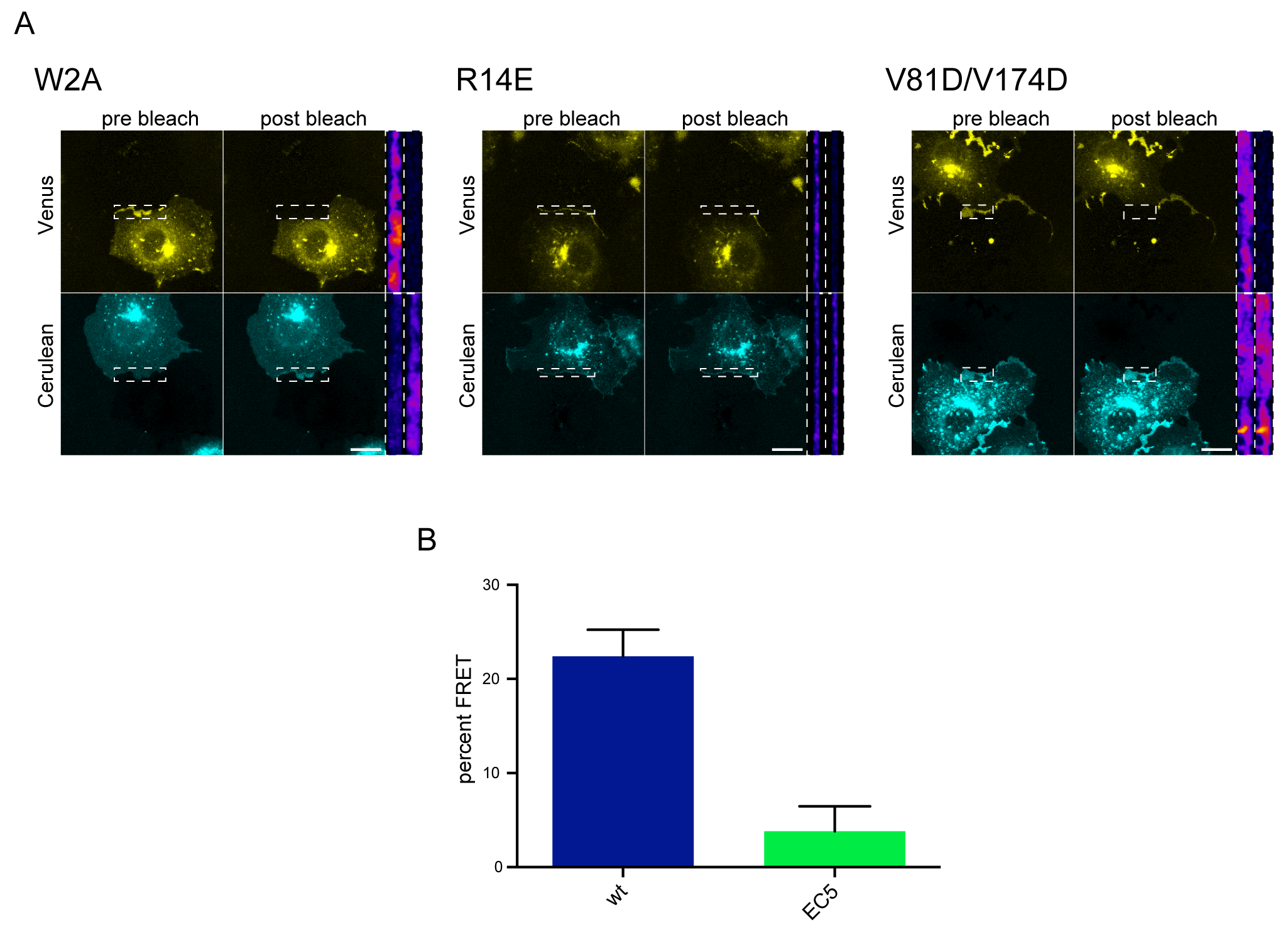

Supplement: Figure S1 — Examples of acceptor bleach experiments for mutants and analysis of control acceptor bleach experiments. (A) Examples for acceptor bleach experiments with COS7-cells expressing either N-cadherin-FP-W2A (strand-swap mutant), -R14E (X-dimer mutant) or -V81D/V174D (cis mutant). The upper two images show the Venus-channel before and after the bleaching of the Venus signal in the junction (boxed region). The Cerulean channel is shown in the lower two images. Bleaching of the Venus fluorescence leads to a dequenching of the FRET donor Cerulean, which can be observed in the lower two images. An enlargement of the junction (boxed region) is shown next to the images. Scale bars = 20 µm. (B) Quantitative comparison of the acceptor bleach experiments for N-cadherin-WT with the insertion of the fluorescent protein within EC2 (WT) or close to EC5 (EC5). The bars represent the mean ± SEM. The mean of the WT (n = 8) is significantly increased compared to EC5 (n = 8, p = 0.0003, unpaired t-test, *** ). (TIF) [file pone.0081517.s001.tif]

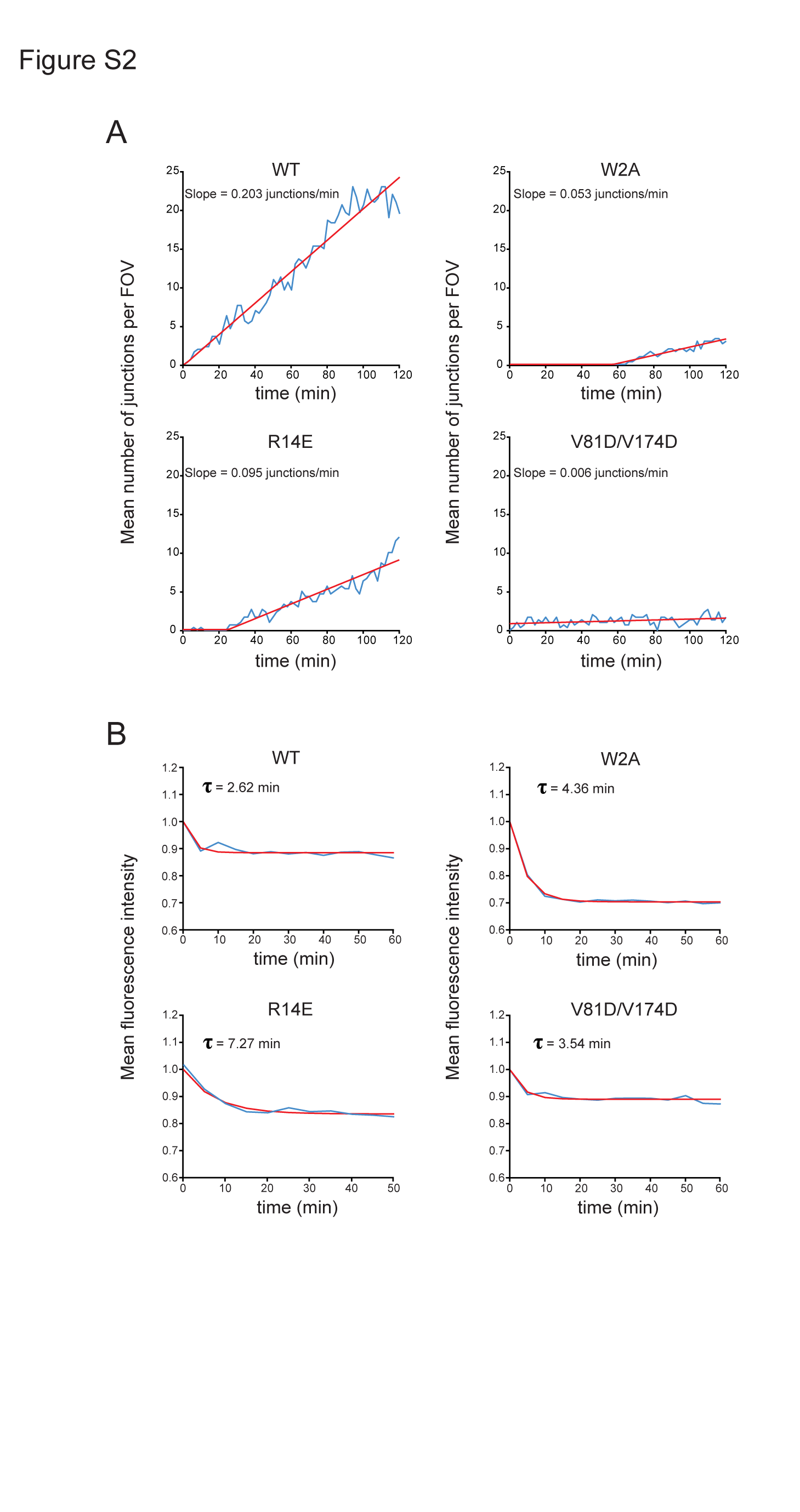

Supplement: Figure S2 — Curve fitting for junction assembly and disassembly. (A) A linear function following a constant baseline was fit to the junction assembly data and the slope was used to determine the rate of junction assembly. WT has the fastest rate of junction formation, followed by R14E, W2A and V81D/V174D. (B) An exponential fit for temporal dynamics of disassembly was performed from the time point of BAPTA addition. The fit for R14E was started 10 min after BAPTA addition, the time point at which the decay started. (TIF) [file pone.0081517.s002.tif]

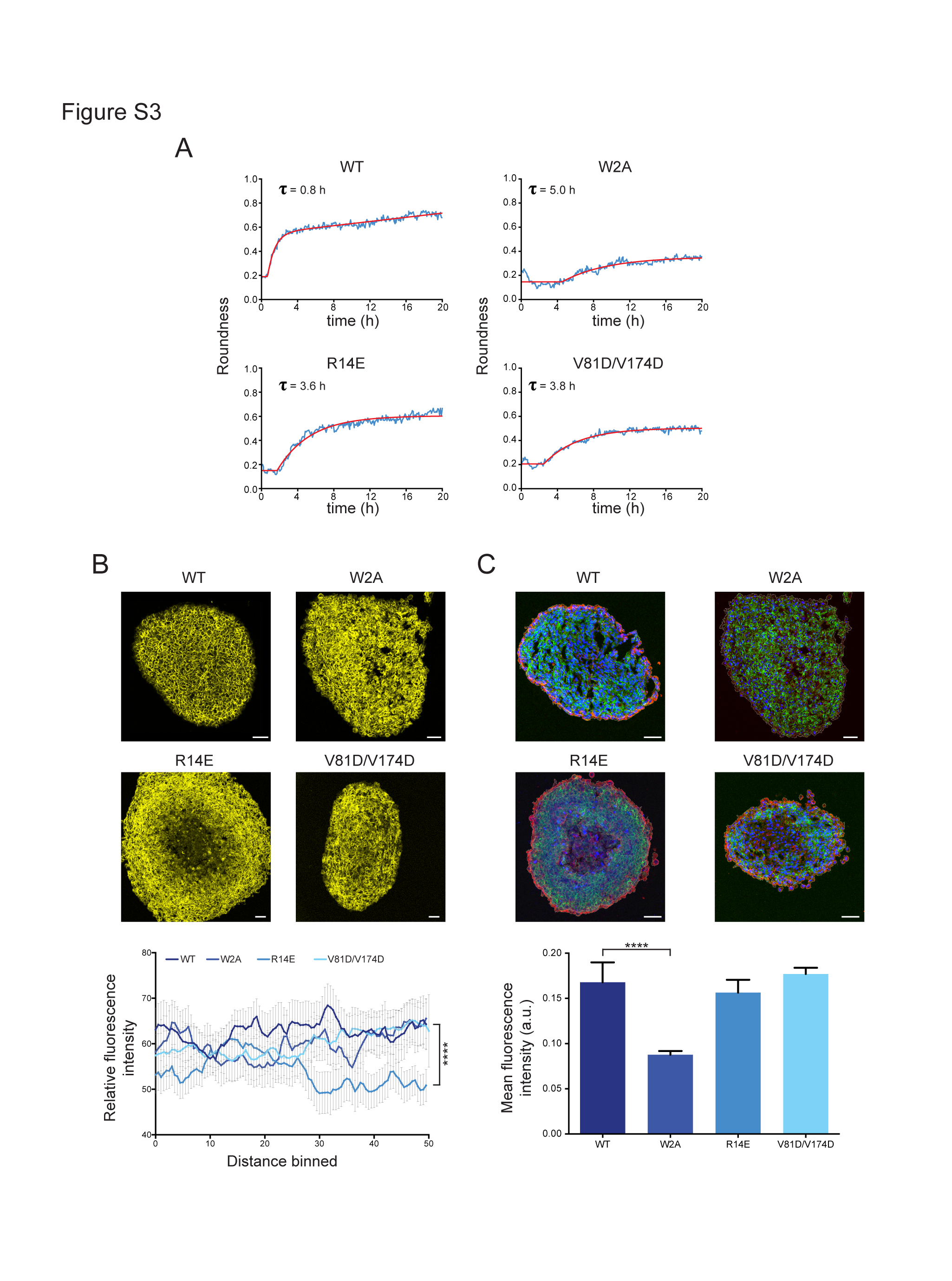

Supplement: Figure S3 — Curve fitting for spheroid formation and analysis of cryo-sections. (A) A sum of two exponentials – fast and slow – following a constant baseline were fit to the roundness data up to 20 h. The tau is displayed for the fast exponential. WT has the fastest rate of spheroid formation, followed by R14E, V81D/V174D and W2A. (B) 10 µm cryo-sections of the spheroids were imaged with 20x 0.8 N.A. Plan-Apochromat objective lens. Using a 99-pixel wide line, each section was straightened along 3 axes, distance binned, and relative fluorescence intensity profiles were plotted. Comparing the intensity profiles between bins 30-50 for all the mutants, only R14E was significantly different compared to WT. (Kruskall-Wallis ANOVA with Dunn’s multiple comparison test, P < 0.05). Error bars indicate SEM. n(WT) = 18, n(W2A) = 20, n(R14E) = 25, n(V81D/V174D) = 18 straightened sections. (C) The cryo-sections were immuno-labeled using an anti-laminin antibody. Using automated detection of laminin on the periphery of the sections, the mean fluorescence intensity of laminin staining was measured. The mean fluorescence intensity of W2A was significantly lower than WT (Mann-Whitney U test, P < 0.0001). Error bars indicate SEM. n(WT) = 7, n(W2A) = 5, n(R14E) = 6, n(V81D/V174D) = 7 spheroids. Scale bars (B) and (C) = 50 µm. (TIF) [file pone.0081517.s003.tif]

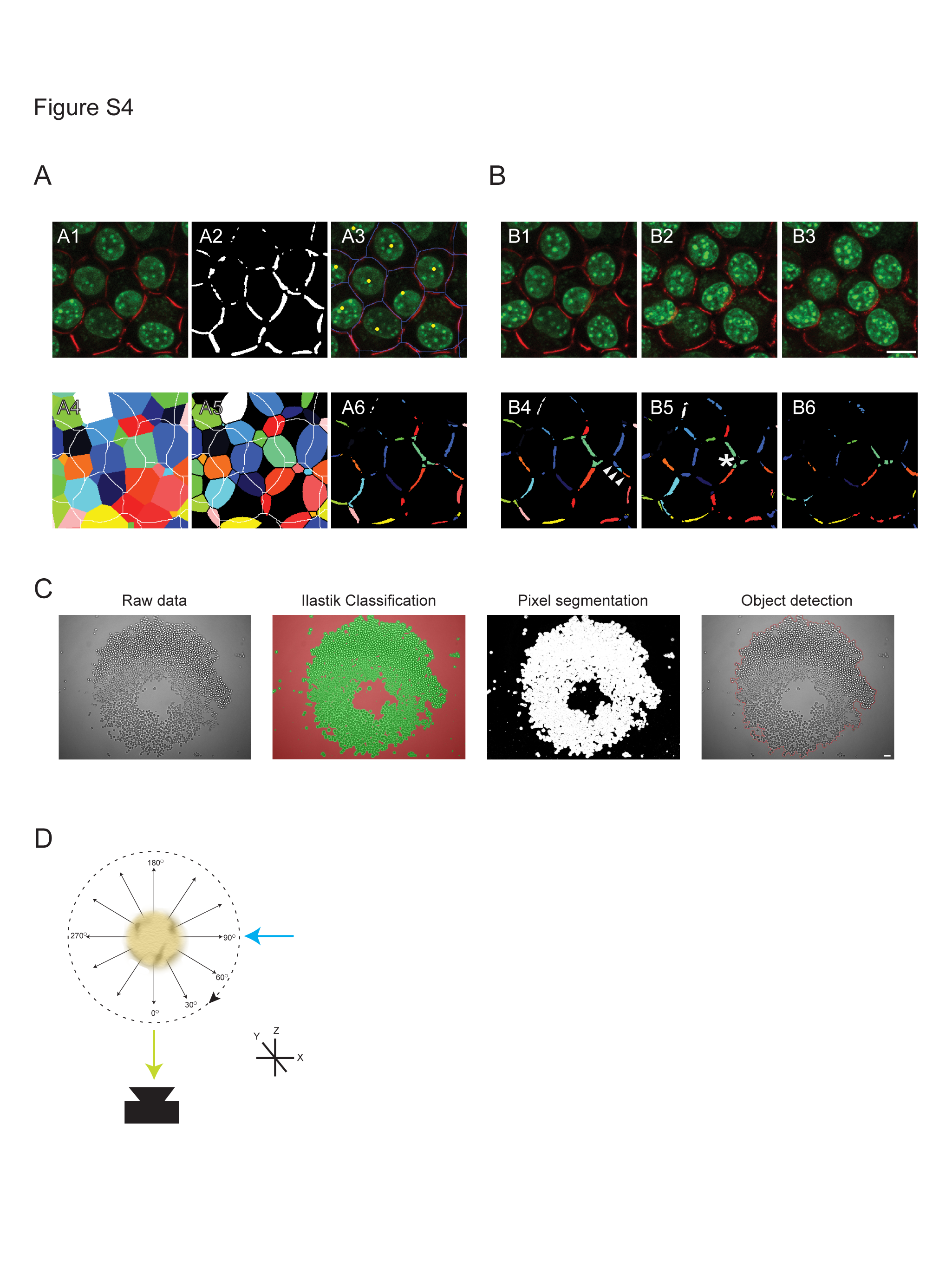

Supplement: Figure S4 — Analysis pipeline and 3D imaging illustration. (A) Identification of junction location across time series. (A1) Maximum intensity projection of two central images of z-stack (red: Venus, green: Hoechst) at time point -15 min. (A2) Threshold mask of Venus channel. (A3) Cell centers (yellow dots) calculated based on a watershed segmentation (blue lines). The watershed was calculated in 3D (x,y,t), so that the ‘identity’ of the cells was preserved across the whole time series. (A4) Two-closest neighbors’ segmentation: Each color patch represents the pixels that share the same pair of cells as their closest neighbors (based on the cells’ centers shown in A3). (A5) Masking segmentation based on distance from the closest cell center. (A6) Combination of the threshold mask (A2) with the two-closest neighbor segmentation (A5). Since cell identity is preserved across frames, the junction identity is too, making it possible to track junctions even when they are discontinuous in space and/or time. (B) Examples of segmentation across time. The top row (B1-B3) shows the fluorescence images (similar to A1) at time points 0, 15 and 30 min. The bottom row (B4-B6) shows the corresponding segmentation (similar to A6). Note that junctions preserve identity (color) across frames. Segmentations that do not represent individual (e.g. star in B5) or complete (e.g. arrow heads in B4) junctions were excluded from further analysis. (C) Identification of spheroid formation over time. Raw data shows the image as it was acquired. An Ilastik classifier was used to define two classes – spheroid (green) and background (red). The pixel segmentation based on the classifier followed by object detection based on the size of spheroid-classified pixels. (D) Imaging the spheroids with mDSLM. The spheroid (yellow) was illuminated (blue arrow) from the side and the detection was done at a 90° angle (green arrow). The spheroid was rotated 12 times in steps of 30° as illustrated. (TIF) [file pone.0081517.s004.tif]
