# Supplementary material for: Role of N-Cadherin cis and trans Interfaces in the Dynamics of Adherens Junctions in Living Cells
Source: PLoS One. 2013 Dec 2;8(12):e81517. doi: 10.1371/journal.pone.0081517 (PMC3847041; doi:10.1371/journal.pone.0081517)
Supplement: Methods S1 — Supporting materials and methods. (DOC) [file pone.0081517.s005.doc]

**Supporting materials and methods**

**Cloning and site-directed mutagenesis**

The generation of the mouse N-cadherin-GFP plasmid by an *in vitro* transposition assay with the insertion of the fluorescent protein at position 151 of the mature protein has been described before (Ki*m et* al, 2011). A large fragment of the coding region of N-cadherin was subcloned via the restriction sites *Kpn*I (nt 2324 of N-cadherin coding region) and *Xba*I (upstream of N-cadherin coding region) into the pBluescriptIISK(-) (resulting plasmid was named pBSKII-NCad-part). A smaller fragment (273-920 of the coding region of N-cadherin) was subcloned into the pEGFP-C1 vector via the restriction sites *Hind*III and *Bam*HI. This plasmid was used as a template for site-directed mutagenesis using the QuickChange II Site-Directed Mutagenesis Kit (Agilent Technologies). The primers 5’-atcaacttgccagaaaactccgagggaccctttcctcaagagctt-3’ (sense) and 5’-aagctcttgaggaaagggtccctcggagttttctggcaagttgat-3’ (antisense) were used for introduction of the point mutation R14E. The mutagenesis reaction was performed according to the manufacturer´s protocol. The mutated DNA fragment was cloned back into pBSKII-NCad-part and then in a second step back into the pCXN2-NCad construct. The mutations V81D and V174D targeting the *cis* interface were consecutively introduced into pBSKII-NCad-part using the primers 5'-cacttgagagcacatgcagatgacatcaatggcaatcaag-3' (sense) and 5'-cttgattgccattgatgtcatctgcatgtgctctcaagtg-3' (antisense) for V81D and 5'-ggggacatcatcactgatgcagctggtctggatc-3' (sense) and 5'-gatccagaccagctgcatcagtgatgatgtcccc-3' (antisense) for V174D. The coding region of EGFP was exchanged with the coding regions of the fluorescent proteins Venus and Cerulean via the restriction site *Asc*I.

**Cell culture and setup of FRET assay**

COS7 cells and L cells were obtained from ATCC and were cultured in a humidified atmosphere at 37°C and 5% CO_2_ using DMEM GlutaMAX (Gibco) supplemented with 10% fetal calf serum (Gibco) and 1 mM sodium pyruvate (Gibco).

COS7 cells grown to confluency in a T75-flask were trypsinized and transfected in suspension with 6 µg of DNA for N-cadherin-Venus or N-cadherin-Cerulean using XtremeGENE9 (Roche Applied Science). Cells were distributed to three wells of a 6-well plate and incubated at 37°C and 5% CO_2_ for 24 h. Cells were detached from the wells by incubation in DPBS containing 1.5 mM EDTA (Gibco) at 37°C for 5 min. Cells individually transfected with N-cadherin-Venus and N-cadherin-Ceruelan encoding plasmid DNA were mixed and spun down at 1000 rpm for 5 min at room temperature. After resuspension in regular medium, cells were plated at high densities on MatTek dishes (35 mm glass bottom dishes, precoated with Matrigel) and incubated for 24-48 h at 37°C and 5% CO_2_. Medium was exchanged to phenolred-free DMEM (Gibco) containing 10% fetal calf serum, 1 mM sodium pyruvate and additional 2 mM CaCl_2_. Before imaging, medium was exchanged with prewarmed Hibernate A (BrainBits, LLC).

**Generation of stable L cell lines**

Monoclonal L cell lines stably expressing the NCad-Venus mutants were generated by limited dilution. Cells were plated in 24-well plates and transfected one day after plating at a confluency of 60-80%. For transfection, 100 µl RPMI (Gibco) were mixed with 6 µl XtremeGENE9 (Roche Applied Sciences) and 2 µg plasmid DNA, followed by an incubation at room temperature for 30 min. 1 µl of combiMag (OZBiosciences) was added and the reaction was incubated for another 20 min at room temperature. 1.4 ml of complete medium was added and then 500-750 µl of this transfection mix were added to 2-3 wells of cells. The 24-well plate was placed on a magnetic plate and incubated for 20 min at 37°C. The plate was then removed from the magnetic plate and incubated at 37°C for 24h. Cells were trypsinized by addition of 100 µl TrypLE (Gibco) and incubation at 37°C for 5 min. The cell suspension was transferred to a 6-well plate containing 2 ml complete medium supplemented with 600 µg/ml G418 (Gibco) in order to start selection for transfected cells revealing resistance to G418. The medium was changed every 2 days and cells were grown until individual colonies appeared (1-2 weeks). Cells were trypsinized by addition of 400 µl TrypLE and incubation at 37°C for 5 min. 1.6 ml complete medium were added and the cell suspension was centrifuged at 1000 rpm for 5 min. Cells were resuspended in complete medium, diluted to a final concentration of 2 cells/ml and plated in a 96-well plate (100 µl/well). Wells were checked for single cells 1-2days after plating. Individual cell clones were grown for 8-14 days and then further expanded in 24-well plates. Expression was checked by Western blot (primary antibody: mouse anti-N-cadherin, BD Biosciences) and positive clones were further subjected to analysis by fluorescence microscopy. Clones with similar expression levels and proper membrane localization were chosen for further analyses.

**Image analyses**

*Acceptor bleach FRET*

Unmixed images were further processed using ImageJ (National Institute of Health). Images were converted to 8bit images, the background was subtracted (rolling ball 50) and they were smoothened (replacement of each pixel with the average of its 3x3 neighborhood). The junctional area was straightened and the FRETcalc v3.0 plug-in was applied to the straightened area. The bleached threshold was set at 50%, the max bleached threshold at 100% and the FRET thresholds at -100 and 100. Pseudocolored spatial FRET maps (0 corresponds to 0% FRET and 255 corresponds to 100% FRET), a histogram showing the distribution of the FRET values obtained for individual pixels and the average FRET of all pixels were obtained as output. The equation E = (E_Cerulean post_ – E_Cerulean pre_) / E_Cerulean post_ was applied by the plugin in order to calculate the FRET efficiencies for individual pixels.

*Ratiometric FRET*

The average intensities of the ROI for the two channels, Venus and Cerulean, were obtained over time. The average fluorescence intensity of both channels for the last 50 images before BAPTA addition was calculated and all values were normalized to this value. The ratio of the normalized Venus and Cerulean values was calculated for each time point. The average for the last 10 images before BAPTA addition and the following 20 images after BAPTA addition were calculated and these values were named “pre FRET” and “post FRET”.

*Junction assembly*

For data analysis, maximum intensity projections were cropped to 355 x 324 pixels in ImageJ (National Institutes of Health). From these cropped images, a blind manual count was used to determine the number of junctions formed over time, where a junction was defined as an increase in mean fluorescence intensity at the point where the membranes of two neighboring cells met. The junction lifetime was calculated between 60 and 120 minutes for all mutants as the number of minutes a single junction was present and was represented as a percent where 100% corresponds to 1h.

*Junction disassembly*

A custom MATLAB (MathWorks, Inc.) script was used to analyze the mean fluorescence intensity of the junctions. The original images were cropped to 512 x 512 pixels and the two z-planes with the cells in focus were combined (maximum projection) (Fig. S4 A1). The junctions were detected by thresholding the Venus fluorescence using an algorithmic threshold (Otsu’s method) determined from the first stack of images acquired (Fig. S4 A2). In order to track individual junctions across frames, each frame was segmented by assigning each pixel to its two closest somata (using the Hoechst channel) (Fig. S4 A4). Soma positions were determined using Gaussian blur (width 3 µm) followed by 3D watershed and frame-wise calculation of center of gravity on the resulting segmentation (Fig. S4 A3). This was manually corrected to make sure a cell maintained its identity throughout the stack. Soma positions and the location of the watershed-lines were additionally used to refine the junction detection by excluding regions outside a certain radius (Fig. S4 A5). The junctions that were identified by the script (Fig. S4 A6 and 4 B1-B6) were further selected manually for the analysis in order to eliminate false positives. The data were normalized to the mean fluorescence intensity at the time when the BAPTA was added. All graphical representations and statistics were done in GraphPad Prism 6.

*Spheroid formation*

Only wells with a spheroid in the field of view for the entire stack were used for data analyses. To determine the roundness of a spheroid, an automated pipeline was developed in CellProfiler (Carpente*r et* al, 2006). After loading in the images, the “classify pixels” module was used to perform pixel classification using Ilastik (Kreshu*k et* al, 2011). The Ilastik classifier was used to segment the images. Two classes were defined – spheroid or background – and the classifier was trained on a small set of images before applying it to the large dataset. After pixel segmentation based on a probability of a pixel representing a spheroid, objects were detected based on size with a typical diameter between 150 to 100,000 spheroid-classified pixels (Fig. S4 C). A manual intensity threshold of 0.3 was applied on the probability maps and the size and shape of the objects was measured. The roundness of an object is given by:

$$Roundness=\frac{4\pi Area}{Perimeter^{2}}$$

Roundness scores range from 0 to 1 where a straight line is 0 while a perfect circle is 1. The data were smoothed using a rolling average of 5 and a statistical analysis was done in GraphPad Prism 6.

*mDSLM*

In order to 3D reconstruct the multiview images, at first, a deconvolution step was performed on all images using Fiji (Schindeli*n et* al, 2012) to remove the blurring effect due to light from out-of-focus structures. The images were then imported in to Amira 5.4.3 (FEI Visualization Sciences Group) for alignment and merging of the various angles. All angles were aligned using the multi planar viewer with the normalized mutual information metric and extensive direction optimizer. Once the images were aligned, they were merged using the arithmetic module according to the expression $a+b$ where *a* = angle 01 and *b* = angle 02, followed by *a* = angle 01 + 02 and *b* = angle 03 and so on. The VolRen module was used to visualize the spheroid in 3D.
